# Accurate predictions on small data with a tabular foundation model

**DOI:** 10.1038/s41586-024-08328-6

**Published:** 2025-01-08

**Authors:** Noah Hollmann, Samuel Müller, Lennart Purucker, Arjun Krishnakumar, Max Körfer, Shi Bin Hoo, Robin Tibor Schirrmeister, Frank Hutter

**Affiliations:** 1 Machine Learning Lab, University of Freiburg, Freiburg, Germany; 2 Computational Medicine, Berlin Institute of Health at Charité, Universitätsmedizin Berlin, Berlin, Germany; 3 Prior Labs, Freiburg, Germany; 4 Neuromedical AI Lab, Department of Neurosurgery, Medical Center - University of Freiburg, Faculty of Medicine, University of Freiburg, Freiburg, Germany; 5 Medical Physics, Department of Diagnostic and Interventional Radiology, Medical Center - University of Freiburg, Faculty of Medicine, University of Freiburg, Freiburg, Germany; 6 ELLIS Institute Tübingen, Tübingen, Germany

**Keywords:** Scientific data, Computer science, Software, Statistics, Computational science

## Abstract

Tabular data, spreadsheets organized in rows and columns, are ubiquitous across scientific fields, from biomedicine to particle physics to economics and climate science^1,2^. The fundamental prediction task of filling in missing values of a label column based on the rest of the columns is essential for various applications as diverse as biomedical risk models, drug discovery and materials science. Although deep learning has revolutionized learning from raw data and led to numerous high-profile success stories^3–5^, gradient-boosted decision trees^6–9^ have dominated tabular data for the past 20 years. Here we present the Tabular Prior-data Fitted Network (TabPFN), a tabular foundation model that outperforms all previous methods on datasets with up to 10,000 samples by a wide margin, using substantially less training time. In 2.8 s, TabPFN outperforms an ensemble of the strongest baselines tuned for 4 h in a classification setting. As a generative transformer-based foundation model, this model also allows fine-tuning, data generation, density estimation and learning reusable embeddings. TabPFN is a learning algorithm that is itself learned across millions of synthetic datasets, demonstrating the power of this approach for algorithm development. By improving modelling abilities across diverse fields, TabPFN has the potential to accelerate scientific discovery and enhance important decision-making in various domains.

## Abstract (web summary)

Tabular Prior-data Fitted Network, a tabular foundation model, provides accurate predictions on small data and outperforms all previous methods on datasets with up to 10,000 samples by a wide margin.

## Main

Throughout the history of artificial intelligence, manually created algorithmic components have been replaced with better-performing end-to-end learned ones. Hand-designed features in computer vision, such as SIFT (Scale Invariant Feature Transform)^10^ and HOG (Histogram of Oriented Gradients)^11^, have been replaced by learned convolutions; grammar-based approaches in natural language processing have been replaced by learned transformers^12^; and the design of customized opening and end-game libraries in game playing has been superseded by end-to-end learned strategies^3,13^. Here we extend this end-to-end learning to the ubiquitous domain of tabular data.

The diversity of tabular data sets them apart from unprocessed modalities such as text and images. While in language modelling for example the meaning of a word is consistent across documents, in tabular datasets the same value can mean fundamentally different things. A drug discovery dataset, for example, might record chemical properties, whereas another dataset in materials science might document thermal and electric properties. This specialization leads to a proliferation of smaller, independent datasets and associated models. To illustrate, on the popular tabular benchmarking website openml.org, 76% of the datasets contain less than 10,000 rows at the time of writing.

Deep learning methods have traditionally struggled with tabular data, because of the heterogeneity between datasets and the heterogeneity of the raw data itself: Tables contain columns, also called features, with various scales and types (Boolean, categorical, ordinal, integer, floating point), imbalanced or missing data, unimportant features, outliers and so on. This made non-deep-learning methods, such as tree-based models, the strongest contender so far^14,15^.

However, these traditional machine learning models have several drawbacks. Without substantial modifications, they yield poor out-of-distribution predictions and poor transfer of knowledge from one dataset to another^16^. Finally, they are hard to combine with neural networks, as they do not propagate gradients.

As a remedy, we introduce TabPFN, a foundation model for small- to medium-sized tabular data. This new supervised tabular learning method can be applied to any small- to moderate-sized dataset and yields dominant performance for datasets with up to 10,000 samples and 500 features. In a single forward pass, TabPFN significantly outperforms state-of-the-art baselines on our benchmarks, including gradient-boosted decision trees, even when these are allowed 4 h of tuning, a speedup of 5,140× (classification) and 3,000× (regression). Finally, we demonstrate various foundation model characteristics of TabPFN, including fine-tuning, generative abilities and density estimation.

## Principled in-context learning

TabPFN leverages in-context learning (ICL)^17^, the same mechanism that led to the astounding performance of large language models, to generate a powerful tabular prediction algorithm that is fully learned. Although ICL was first observed in large language models, recent work has shown that transformers can learn simple algorithms such as logistic regression through ICL^18–21^. Prior-data Fitted Networks (PFNs) have shown that even complex algorithms, such as Gaussian Processes and Bayesian Neural Networks, can be approximated with ICL^22^. ICL enables us to learn a wider space of possible algorithms, including cases for which a closed-form solution does not exist.

We build on a preliminary version of TabPFN^23^, which demonstrated the applicability of in-context-learning^17^ for tabular data in principle but had many limitations that rendered it inapplicable in most cases. Based on a series of improvements, the new TabPFN scales to 50× larger datasets; supports regression tasks, categorical data and missing values; and is robust to unimportant features and outliers.

The key idea behind TabPFN is to generate a large corpus of synthetic tabular datasets and then train a transformer-based^12^ neural network to learn to solve these synthetic prediction tasks. Although traditional approaches require hand-engineered solutions for data challenges such as missing values, our method autonomously learns effective strategies by solving synthetic tasks that include these challenges. This approach leverages ICL as a framework for exemplar-based declarative programming of algorithms. We design desired algorithmic behaviour by generating diverse synthetic datasets that demonstrate the desired behaviour and then train a model to encode an algorithm that satisfies it. This shifts the algorithm design process from writing explicit instructions to defining input–output examples, opening up possibilities for creating algorithms in various domains. Here, we apply this approach to the high-impact field of tabular learning, generating a powerful tabular prediction algorithm.

Our ICL approach differs fundamentally from standard supervised deep learning. Usually, models are trained per dataset, updating model parameters on individual samples or batches according to hand-crafted weight-updating algorithms, such as Adam^24^. At inference time, the learned model is applied to test samples. By contrast, our approach is trained across datasets and is applied to entire datasets at inference time rather than individual samples. Before being applied to real-world datasets, the model is once pre-trained on millions of synthetic datasets representing different prediction tasks. At inference time, the model receives an unseen dataset with both labelled training and unlabelled test samples and performs training and prediction on this dataset in a single neural network forward pass.

**Fig. 1:**
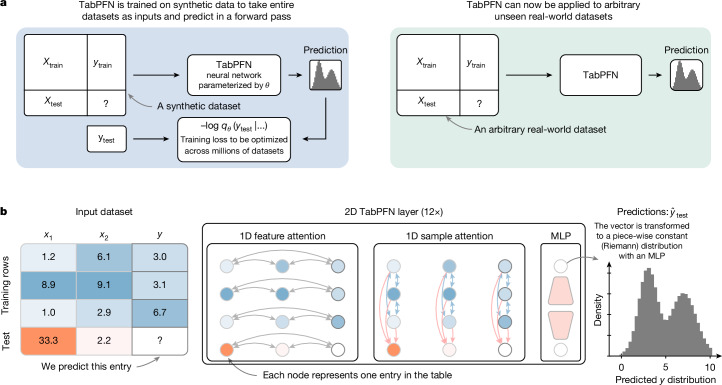
Overview of the proposed method. **a**, The high-level overview of TabPFN pre-training and usage. **b**, The TabPFN architecture. We train a model to solve more than 100 million synthetic tasks. Our architecture is an adaptation of the standard transformer encoder that is adapted for the two-dimensional data encountered in tables.

**Fig. 2:**
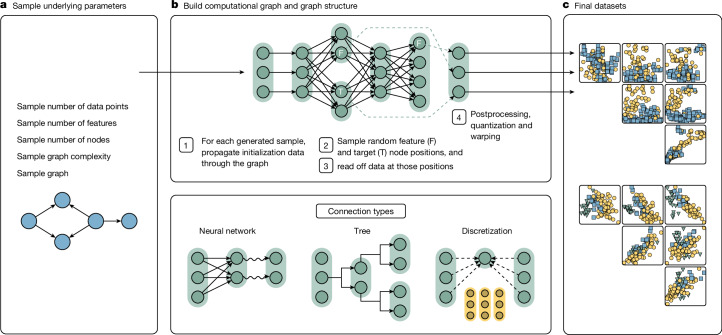
Overview of the TabPFN prior. **a**, For each dataset, we first sample high-level hyperparameters. **b**, Based on these hyperparameters, we construct a structural causal model that encodes the computational function generating the dataset. Each node holds a vector and each edge in the computational graph implements a function according to one of the connection types. In step 1, using random noise variables we generate initialization data, which is fed into the root nodes of the graphs and propagated through the computational graph for each to-be-generated sample. In step 2, we randomly sample feature and target node positions in the graph, labelled F and T, respectively. In step 3, we extract the intermediate data representations at the sampled feature and target node positions. In step 4, we post-process the extracted data. **c,** We retrieve the final datasets. We plot interactions of feature pairs and the node colour represents the class of the sample.

Figures 1 and 2 outline our approach:

1. Data generation: we define a generative process (referred to as our prior) to synthesize diverse tabular datasets with varying relationships between features and targets, designed to capture a wide range of potential scenarios that our model might encounter. We sample millions of datasets from the generative process. For each dataset, a subset of samples has their target values masked, simulating a supervised prediction problem. Further details of our prior design are shown in the section ‘Synthetic data based on causal models’.
2. Pre-training: we train a transformer model, our PFN, to predict the masked targets of all synthetic datasets, given the input features and the unmasked samples as context. This step is done only once during model development, learning a generic learning algorithm that can be used to predict any dataset.
3. Real-world prediction: the resulting trained model can now be applied to arbitrary unseen real-world datasets. The training samples are provided as context to the model, which predicts the labels of these unseen datasets through ICL.

Our approach also has a theoretical foundation as described in ref. ^22^. It can be viewed as approximating Bayesian prediction for a prior defined by the synthetic datasets. The trained PFN will approximate the posterior predictive distribution $p({\widehat{{\bf{y}}}}_{{\rm{test}}}| {{\bf{X}}}_{{\rm{test}}},{{\bf{X}}}_{{\rm{train}}},{{\bf{y}}}_{{\rm{train}}})$  and thus return a Bayesian prediction for the specified distribution over artificial datasets used during PFN pre-training.

## An architecture designed for tables

The transformer architecture is currently the favoured architecture for flexible deep learning and foundation models^4,5^. Transformer models work on sequences and combine information between sequence items using so-called attention mechanisms^25^, allowing them to effectively capture long-range dependencies and learn complex relationships in data. Although transformer-based models can be applied to tabular data^26,27^, TabPFN addresses two key limitations inherent to them. First, as transformers are designed for sequences, they treat the input data as a single sequence, not using the tabular structure. Second, machine learning models are often used in a fit-predict model, in which a model is fitted on the training set once and then reused for multiple test datasets. Transformer-based ICL algorithms, however, receive train and test data in a single pass and thus perform training and prediction at once. Thus, when a fitted model is reused, it has to redo computations for the training set.

To better use the tabular structure, we propose an architecture that assigns a separate representation to each cell in the table, inspired by refs. ^22,28^. Our architecture, visualized in Fig. 1b, uses a two-way attention mechanism, with each cell attending to the other features in its row (that is, its sample) and then attending to the same feature across its column (that is, all other samples). This design enables the architecture to be invariant to the order of both samples and features and enables more efficient training and extrapolation to larger tables than those encountered during training, in terms of both the number of samples and features.

To mitigate repeating computations on the training set for each test sample in a fit-predict setting, our model can separate the inference on the training and test samples. This allows us to perform ICL on the training set once, save the resulting state and reuse it for multiple test set inferences. On datasets with 10,000 training samples and 10 features, our optimized train-state caching results in inference speedups of around 300× on CPU (from 32 s to 0.1 s) and 6× on GPU. With 10× more features (100), the speedups increase to 800× on CPU and 30× speedup on GPU. These measurements focus solely on the core inference process, excluding pre-processing and ensembling steps detailed in the section ‘Inference details’. The lower speedups on GPUs are because of an underutilization of their massively parallel architecture.

We further optimize the memory and compute requirements of the architecture by computing layer norms in half-precision, using flash attention^29^, activation checkpointing and sequential computation of the state. Our optimizations reduce the memory requirements by a factor of four, resulting in less than 1,000 bytes per cell. This enables the prediction on datasets with up to 50 million cells (for example, 5 million rows × 10 features) on a single H100 GPU.

For regression tasks, we use a piece-wise constant output distribution, following refs. ^22,30^, which allows our models to predict a probability distribution of target values instead of a single value, including, for example, bimodal distributions.

## Synthetic data based on causal models

The performance of TabPFN relies on generating suitable synthetic training datasets that capture the characteristics and challenges of real-world tabular data. To generate such datasets, we developed an approach based on structural causal models (SCMs)^31^. SCMs provide a formal framework for representing causal relationships and generative processes underlying the data. By relying on synthetic data instead of large collections of public tabular data, we avoid common problems of foundational models, such as privacy and copyright infringements, contaminating our training data with test data^32^ or limited data availability.

As shown in Fig. 2, our generative pipeline first samples high-level hyperparameters, such as dataset size, number of features and difficulty level, to govern the overall properties of each synthetic dataset. Guided by these hyperparameters, we construct a directed acyclic graph specifying the causal structure underlying the dataset.

To generate each sample within a dataset, we propagate randomly generated noise, called our initialization data, through the root nodes of the causal graph. This initialization data are generated by sampling from a random normal or uniform distribution with varying degrees of non-independence between samples, see section ‘Initialization data sampling’. As these data traverse the edges of the computational graph, we apply a diverse set of computational mappings: small neural networks with linear or nonlinear activations (for example, sigmoid, ReLU (rectified linear unit), modulo, sine), discretization mechanisms for generating categorical features and decision tree structures to encode local, rule-based dependencies. At each edge, we add Gaussian noise, introducing uncertainty into the generated data. We save the intermediate data representations at each node to be retrieved later. See section ‘Computational edge mappings’ for details.

After traversing the causal graph, we extract the intermediate representations at the sampled feature and target nodes, yielding a sample consisting of feature values and an associated target value.

By incorporating various data challenges and complexities into the synthetic datasets, we create a training ground that allows TabPFN to develop strategies for handling similar issues in real-world datasets. For instance, consider the case of missing values, commonly present in tabular data. By exposing TabPFN to synthetic datasets with varying patterns and fractions of missing values in our synthetic data generation process, the model learns effective ways of handling missing values that generalize to real-world datasets. We apply post-processing techniques to further enhance the realism and challenge the robustness of the learned prediction algorithms. This includes warping with the Kumaraswamy distribution^33^, introducing complex nonlinear distortions and quantization mimicking discretized features. See section ‘Post-processing’ for details.

Through this generative process, we created a massive corpus of around 100 million synthetic datasets per model training, each with a unique causal structure, feature types and functional characteristics.

## Qualitative analysis

**Fig. 3:**
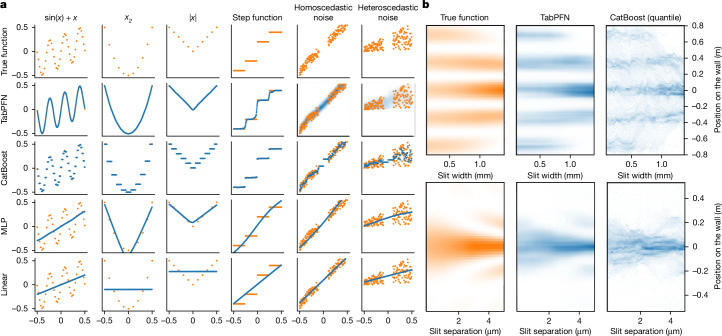
The behaviour of TabPFN and a set of baselines on simple functions. In all plots, we use orange for the ground truth and blue for model predictions. **a**, Each column represents a different toy function, each having a single feature (along the *x*-axis) and a target (along the *y*-axis). TabPFN can model a lot of different functions, including noisy functions. **b**, TabPFN can model distributions over outputs out of the box, which is exemplified by predicting the light intensity pattern in a double-slit experiment after observing the positions of 1,000 photons.

We first analyse the behaviour of TabPFN on toy problems to build intuition and disentangle the impact of various dataset characteristics. As regression problems are easier to visualize, we focus on these in our qualitative analysis. In Fig. 3a, we compare TabPFN with a diverse set of standard predictors, with all methods using default settings.

Linear (ridge) regression can naturally model only linear functions, leading to simple and interpretable predictions but catastrophic failure on many of the toy functions. Multilayer perceptrons (MLPs)^34^ perform worse on datasets with highly non-smooth patterns^14^. This is especially apparent for the step function. TabPFN, by contrast, models either function type, smooth or non-smooth, out of the box. This includes a good approximation to step functions despite TabPFN being a neural network. CatBoost^9^, representative of tree-based methods, fits only piece-wise constant functions. Although this leads to approximation errors and unintuitive predictions, it avoids catastrophic failures.

The main advantage of TabPFN over all baselines is its inherent ability to model uncertainty at no extra cost. Whereas classical regression methods output a single real-valued prediction, TabPFN returns a target distribution, capturing the uncertainty of predictions. These uncertainty modelling abilities of TabPFN extend beyond simple distributions and can handle complex, multi-modal distributions. Figure 3b shows this by modelling the density of light reaching a detector screen in a double-slit experiment^35^ for different slit distances and widths. In this classic experiment, photons are sent through two slits creating a multi-modal intensity pattern because of the wave-like interference behaviour of light. TabPFN predicts these intricate patterns in just a single forward pass, requiring only 1.2 s. By contrast, traditional methods such as CatBoost require training multiple quantile models at different quantiles and reconstructing the distribution from these predictions. Even after tuning CatBoost specifically for this task, it produced substantially worse predictions compared with TabPFN, see Fig. 3b. With default settings, CatBoost requires 169.3 s and yields further deteriorated results. Qualitatively, we observe that TabPFN is more accurate in predicting very low densities and has fewer artefacts compared with CatBoost.

## Quantitative analysis

We quantitatively evaluate TabPFN on two dataset collections: the AutoML Benchmark^36^ and OpenML-CTR23^37^. These benchmarks comprise diverse real-world tabular datasets, curated for complexity, relevance and domain diversity. From these benchmarks, we use the 29 classification datasets and 28 regression datasets that have up to 10,000 samples, 500 features and 10 classes. We further evaluated additional benchmark suites from refs. ^14,15^, as well as five Kaggle competitions from the Tabular Playground Series.

We compared TabPFN against state-of-the-art baselines, including tree-based methods (random forest^38^, XGBoost (XGB)^7^, CatBoost^9^, LightGBM^8^), linear models, support vector machines (SVMs)^39^ and MLPs^34^.

Evaluation metrics include ROC AUC (area under the receiver operating characteristic curve; One-vs-Rest) and accuracy for classification, and *R*^2^ (coefficient of determination) and negative RMSE (root mean squared error) for regression. Scores were normalized per dataset, with 1.0 representing the best and 0.0 the worst performance with respect to all baselines.

For each dataset and method, we ran 10 repetitions with different random seeds and train–test splits (90% train, 10% test). We tuned hyperparameters using random search with five-fold cross-validation, with time budgets ranging from 30 s to 4 h. All methods were evaluated using eight CPU cores, with TabPFN additionally using a consumer-grade GPU (RTX 2080 Ti; other methods did not benefit from this, see Extended Data Fig. 2d). TabPFN was pre-trained once using eight NVIDIA RTX 2080 GPUs over 2 weeks, allowing for ICL on all new datasets in a single forward pass. These modest computational requirements make similar research accessible to academic labs. For details, refer to the section ‘Detailed evaluation protocol’.

### Comparison with state-of-the-art baselines

**Fig. 4:**
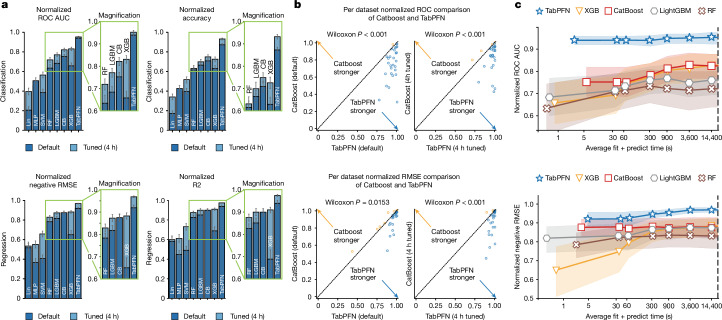
Comparison of TabPFN on our test benchmarks, containing datasets with up to 10,000 samples and 500 features. Performance was normalized per dataset before aggregation using all baselines; intervals represent the 95% confidence interval. Wilcoxon *P* refers to the two-sided Wilcoxon signed-rank test *P* value^54^. **a**, Average performance of the default as well as the tuned versions of TabPFN and our baselines. All methods are tuned for ROC AUC or RMSE, respectively, thus decreasing the representativeness of the secondary metrics. LGBM, LightGBM; MLP, multilayer perceptron; SVM, support vector machines; RF, random forest; CB, CatBoost; XGB, XGBoost; Lin, logistic regression for classification and ridge regression for regression tasks. Plots on the right-hand side show a magnified analysis of the strongest baselines considered. **b**, A per-dataset comparison of TabPFN with its strongest baseline, CatBoost. Each dot is the average score on one dataset. **c**, The impact of hyperparameter tuning for the considered methods. The *x*-axis shows the average time required to fit and predict with the algorithm.

Figure 4a demonstrates the strong out-of-the-box performance of TabPFN compared with tuned and default configurations of XGBoost, CatBoost and a random forest. For classification tasks, TabPFN surpasses CatBoost, the strongest default baseline, by 0.187 (0.939 compared with 0.752) in normalized ROC AUC in the default setting and by 0.13 (0.952 compared with 0.822) in the tuned setting. For regression, TabPFN outperforms CatBoost in normalized RMSE by 0.051 (0.923 compared with 0.872) in the default setting and by 0.093 (0.968 compared with 0.875) in the tuned setting. In Fig. 4b, we show per-dataset comparisons. Although for some datasets CatBoost outperforms TabPFN, TabPFN wins on most of the datasets.

Figure 4c shows how the performance of TabPFN and the baselines improve with more time spent on hyperparameter search. The default of TabPFN, taking 2.8 s on average for classification and 4.8 s for regression, outperforms all baselines, even when tuning them for 4 h—a speedup of 5,140× and 3,000×, respectively. We show comparisons on a larger number of metrics in Extended Data Tables 1 and 2.

As shown in Extended Data Fig. 2, similar to our primary benchmarks, TabPFN substantially outperformed all baselines on the benchmarks of refs. ^14,15^. The benchmark of ref. ^14^ is particularly noteworthy because on this benchmark, tree-based methods were previously found to excel. Moreover, we show in Extended Data Table 6 that default TabPFN outperforms default CatBoost on all five Kaggle competitions with less than 10,000 training samples from the latest completed Tabular Playground Series.

### Evaluating diverse data attributes

**Fig. 5:**
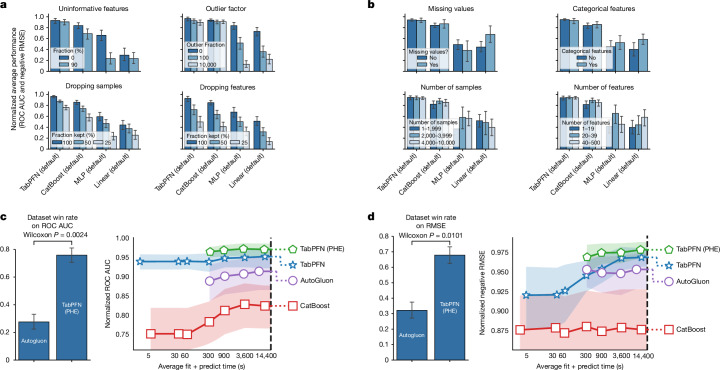
Robustness across datasets and performance comparison with tuned ensembles. **a**, A comparison of modified datasets. We can see that TabPFN is not more vulnerable to the modifications compared with baselines. We also see that TabPFN reproduces the accuracy of CatBoost (default) with only half the training samples provided. Here we normalize scores per dataset (sharing one normalization across all modifications of one experiment) to avoid negative outliers. **b**, We split the test datasets by data characteristics and analyse the performance per subgroup. **c**, Classification performance. Left, the win rate of TabPFN (PHE) against AutoGluon (with one tie excluded); right, the ROC AUC score over time for tuning each method, with the first marker representing the default configuration for the non-ensembling methods. **d**, Regression performance presented as in **c** but using the RMSE metric. Intervals represent the 95% confidence interval and Wilcoxon *P* refers to the two-sided Wilcoxon signed-rank test *P* value^54^.

In Fig. 5a,b, we show the robustness of TabPFN to dataset characteristics that are traditionally hard to handle for neural-network-based approaches^14,23^.

Figure 5a provides an analysis of the performance of TabPFN across various dataset types. First, we add uninformative features (randomly shuffled features from the original dataset) and outliers (multiply each cell with 2% probability with a random number between 0 and the outlier factor). The results show that TabPFN is very robust to uninformative features and outliers, something typically hard for neural networks, as can be seen with the MLP baseline. Second, although dropping either samples or features hurts the performance of all methods, with half the samples TabPFN still performs as well as the next best method using all samples.

**Extended Data Fig. 1:**
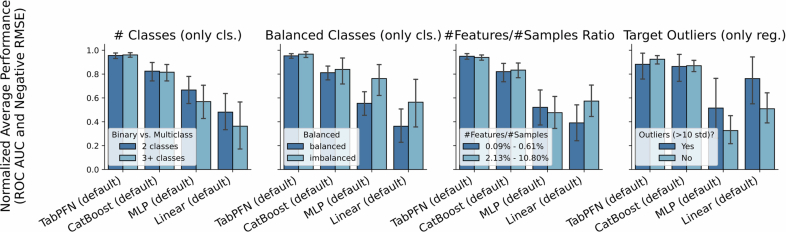
Performance comparison across additional dataset characteristics, extending Fig. 5. This figure shows the relative performance of different methods when datasets are split based on specific attributes. Error bars represent 95% confidence intervals. While performance differences are generally subtle across these splits, the most notable variation is observed for datasets with outliers in the target variable, though confidence intervals still overlap.

In Fig. 5b, we split our test datasets into subgroups and perform analyses per subgroup. We create subgroups based on the presence of categorical features, missing values, number of samples and number of features in the datasets. The sample- and feature-number subgroups are split such that a third of the datasets fall into each group. We can see that none of these characteristics strongly affect the performance of TabPFN relative to the other methods. However, we note that these results should not be taken as evidence that TabPFN scales well beyond the 10,000 samples and 500 features considered here. We show four further ablations in Extended Data Fig. 1.

### Comparison with tuned ensemble methods

We compare the performance of TabPFN with AutoGluon 1.0 (ref. ^40^), which combines various machine learning models, including our baselines, into a stacked ensemble^41^, tunes their hyperparameters and then generates the final predictions using post hoc ensembling (PHE)^42,43^. It thus represents a different class of methods compared with individual baselines.

To assess whether TabPFN can also be improved by a tuned ensemble approach, we introduce TabPFN (PHE). TabPFN (PHE) automatically combines only TabPFN models with PHE and tunes their hyperparameters using a random portfolio from our search space. We detail this approach in the section ‘TabPFN (PHE)’.

Figure 5c–d compares the performance of TabPFN, TabPFN (PHE), AutoGluon and CatBoost. For TabPFN (PHE) and AutoGluon, we start with a minimal budget of 300 s for tuning because AutoGluon otherwise does not reliably return results. In just 2.8 s, TabPFN (default) outperforms AutoGluon for classification tasks, even if AutoGluon is allowed up to 4 h, a 5.140× speedup. TabPFN (PHE) further improves performance leading to an average normalized ROC AUC score of 0.971, compared with 0.939 for TabPFN (default) and 0.914 for AutoGluon. For regression tasks, tuning hyperparameters is more important. Here, TabPFN (PHE) outperforms AutoGluon (allowed 4 h) after its minimal tuning budget of 300 s, a 48× speedup.

## Foundation model with interpretability

Apart from its strong predictive performance, TabPFN exhibits key foundation model abilities, such as data generation, density estimation, learning reusable embeddings and fine-tuning. We showcase these abilities through proof-of-concept experiments on the German Credit Dataset^44^, which contains credit risk information and the mfeat-factors^45^ dataset classifying handwritten digits based on a tabular representation.

**Fig. 6:**
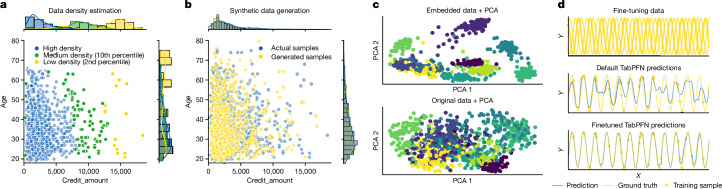
Showcase of the application of TabPFN as tabular foundation model. **a**,**b**, On the German Credit Dataset, we perform data density estimation (**a**) and generation of new synthetic samples (**b**). **c**, We show our learned embeddings are useful representations of each sample on the handwritten digits dataset (mfeat-factors) with different classes forming different clusters. **d**, We demonstrate fine-tuning TabPFN for a specific set of tasks. Fine-tuned on a dataset containing various sine curves (top), we see the model makes more accurate predictions on another sine curve dataset.

TabPFN can estimate the probability density function of numerical features, as shown in Fig. 6a, and the probability mass function of categorical features. Computing the sample densities enables anomaly detection to identify issues such as fraud, equipment failures, medical emergencies or low-quality data.

TabPFN also allows synthesizing new tabular data samples that mimic real-world dataset characteristics as shown in Fig. 6b. This enables applications such as data augmentation or privacy-preserving data sharing^46^.

The architecture of TabPFN yields meaningful feature representations that can be reused for downstream tasks such as data imputation and clustering. We extract and visualize learned embeddings from the mfeat-factors dataset in Fig. 6c, showing improved class separation compared with the raw data on the first two principal components.

Furthermore, we demonstrate the ability of TabPFN to improve performance through fine-tuning on related datasets. Unlike tree-based methods, the neural architecture of TabPFN enables fine-tuning on specific dataset classes. We conduct proof-of-concept experiments using sine curve datasets with varying offsets between fine-tuning and test data. Figure 6d shows an example fine-tuning result. Our analysis across 50 runs (Extended Data Fig. 4) shows that TabPFN successfully transfers knowledge even when labels differ significantly between fine-tuning and test tasks, with performance improving as distributions become more similar. This could, for example, enable fine-tuning for a range of datasets from medical studies to obtain an improved general model for medical diagnosis tasks. For details, refer to section ‘Foundation model abilities’.

Finally, we have developed a methodology to easily interpret the predictions of TabPFN. Interpretability is crucial for building trust and accountability when deploying models in high-stakes domains. We support the computation of feature importance through SHAP^47^ (Shapley Additive Explanations), a game-theoretic approach to explain predictions. SHAP values represent the contribution of each feature to the output of the model. Extended Data Fig. 3 compares the feature importance and impact for logistic regression, CatBoost and TabPFN. TabPFN achieves high accuracy while learning simple, interpretable feature relationships. By contrast, logistic regression is interpretable but less accurate, whereas CatBoost is accurate but qualitatively less interpretable because of complex, non-smooth decision boundaries.

## Conclusion

TabPFN represents a major change in tabular data modelling, leveraging ICL to autonomously discover a highly efficient algorithm that outperforms traditional human-designed approaches on datasets with up to 10,000 samples and 500 features. This shift towards foundation models trained on synthetic data opens up new possibilities for tabular data analysis across various domains.

Potential future directions include scaling to larger datasets^48^, handling data drift^49^, investigating fine-tuning abilities across related tabular tasks^50^ and understanding the theoretical foundations of our approach^51^. Future work could also explore creating specialized priors to handle data types such as time series^52^ and multi-modal data, or specialized modalities such as ECG, neuroimaging data^53^ and genetic data. As the field of tabular data modelling continues to evolve, we believe that foundation models, such as TabPFN, will play a key part in empowering researchers. To facilitate the widespread use of TabPFN, in the section ‘User guide’ we discuss how to use it effectively.

## Methods

### User guide

#### When to use TabPFN

TabPFN excels in handling small- to medium-sized datasets with up to 10,000 samples and 500 features (Fig. 4 and Extended Data Table 1). For larger datasets and highly non-smooth regression datasets, approaches such as CatBoost^9^, XGB^7^ or AutoGluon^40^ are likely to outperform TabPFN.

Although TabPFN provides a powerful drop-in replacement for traditional tabular data models such as CatBoost, similar to these models, it is intended to be only one component in the toolkit of a data scientist. Achieving top performance on real-world problems often requires domain expertise and the ingenuity of data scientists. As for other modelling approaches, data scientists should continue to apply their skills and insights in feature engineering, data cleaning and problem framing to get the most out of TabPFN. We hope that the training speed of TabPFN will facilitate faster iterations in the data science workflow.

#### Limitations of TabPFN

The limitations of TabPFN are as follows: (1) the inference speed of TabPFN may be slower than highly optimized approaches such as CatBoost; (2) the memory usage of TabPFN scales linearly with dataset size, which can be prohibitive for very large datasets; and (3) our evaluation focused on datasets with up to 10,000 samples and 500 features; scalability to larger datasets requires further study.

#### Computational and time requirements

TabPFN is computationally efficient and can run on consumer hardware for most datasets. However, training on a new dataset is recommended to run on a (consumer) GPU as this speeds it up by one to three orders of magnitude. Although TabPFN is very fast to train, it is not optimized for real-time inference tasks. For a dataset with 10,000 rows and 10 columns, our model requires 0.2 s (0.6 s without GPU) to perform a prediction for one sample, whereas CatBoost (default) can do the same in 0.0002 s. In ref. ^55^, further optimizing TabPFN specifically for inference tasks has already been explored, resulting in four times faster inference performance compared with even XGBoost, but so far also reducing predictive quality. Refer to the section ‘Details on the neural architecture’ for details on the memory usage and runtime complexity of TabPFN.

#### Data preparation

TabPFN can handle raw data with minimal pre-processing. If we simply provide the data in a tabular format (NumPy matrix), TabPFN will automatically handle missing values, encode categorical variables and normalize features. Although TabPFN works well out of the box, we can further improve the performance using dataset-specific pre-processing. This can also be partly done automatically with our PHE technique or manually by modifying the default settings. When manually pre-processing data, we should keep in mind that the neural network of TabPFN expects roughly normally distributed features and targets after all pre-processing steps. If we, for example, know that a feature follows a log distribution, it might help to exponentiate it before feeding it to TabPFN. As TabPFN does *z*-normalization of all inputs, scaling does not affect the predictions. As for all algorithms, however, using domain knowledge to combine or remove features can increase performance.

#### Hyperparameter tuning

TabPFN provides strong performance out of the box without extensive hyperparameter tuning (see section ‘Comparison with state-of-the-art baselines’). If we have additional computational resources, we can further optimize the performance of TabPFN using hyperparameter optimization (HPO) or the PHE technique described in the section ‘TabPFN (PHE)’. Our implementation directly provides HPO with random search and PHE.

### Details on the neural architecture

Our architecture is a variation of the original transformer encoder^12^ and the original PFN architecture^22^, but it treats each cell in the table as a separate time position, similar to that in ref. ^28^. Therefore, it can generalize to more training samples as well as features than seen during training.

Figure 1b details our new architecture. All features that go into our architecture are first mapped to floating point values, that is, categoricals are transformed to integers. These values are subjected to *z*-normalization using the mean and standard deviation for each feature separately across the whole training set. These values are now encoded with simple linear encoders. Each layer first has an attention over features, followed by an attention over samples, both of which operate separately on each column or row, respectively. These two sub-layers are followed by an MLP sublayer. Each sublayer is followed by a residual addition and a half-precision layer norm.

We found that encoding groups of features can be even more effective compared with encoding one value per representation. For our hyperparameter search space, we selected six architectures for classification and five for regression. In three of the six classification models and four of the five regression models, including the TabPFN default, a transformer position encodes two features of one example; in others, it represents one value.

Although the inter-feature attention is a classical fully connected attention, our inter-sample attention does not allow the test samples to attend to each other but only to the training data. Therefore, we make sure that the test samples do not influence each other or the training set representations. To allow our model to differentiate features more easily that have the same statistics, for example, two features that have the same entries just in different orders, we use random feature embeddings that we add to all embeddings before the first layer. We generate one embedding per feature by projecting a random vector of one-fourth the size of our embeddings through a learned linear layer and add this to all embeddings representing an instance of that feature.

As the representations of training samples are not influenced by the test set, we cache the keys and values of the training samples to allow splitting training and inference. We use a special variant of multi-query attention for our inter-sample attention from test samples^56^ to save memory when caching representations. In our variant, we use all keys and values for the attention between samples of the training set, but repeatedly use the first key and value for attention from the test samples. This allows caching only one key or value vector pair per cell in the training set that is fed into our inter-sample attention of new test samples.

The compute requirements of this architecture scale quadratically with the number of samples (*n*) and the number of features (*m*), that is *O*(*n*^2^ + *m*^2^), and the memory requirements scale linearly in the dataset size, *O*(*n* ⋅ *m*).

Finally, we found that pre-processing inputs can help performance, thus we can perform *z*-normalization of all inputs across the sample dimension and add an extra input for each cell that indicates whether the input was missing; the input itself is set to 0 in these cases. All inputs are finally linearly encoded into the embedding dimension of TabPFN.

### Details on the causal generative process

An SCM ${\mathcal{G}}:= (Z,{\epsilon })$ consists of a collection *Z* ≔ (*z*_1_, …, *z*_*k*_) of structural assignments (called mechanisms): ${z}_{i}={f}_{i}({z}_{{\rm{PA}}{\mathcal{G}}(i)},{{\epsilon }}_{i})\,,$ where ${\rm{PA}}\,{\mathcal{G}}(i)$ is the set of parents of node *i* (its direct causes) in the underlying directed acyclic graph (DAG) ${\mathcal{G}}$ (the causal graph), *f*_*i*_ is a (potentially nonlinear) deterministic function and *ϵ*_*i*_ is a noise variable. Causal relationships in ${\mathcal{G}}$ are represented by edges pointing from causes to effects^31^. As our prior is a sampling procedure, we can make a lot of choices on, for example, the graph size or complexity. By defining a probability distribution over these hyperparameters in the prior, the posterior predictive distribution approximated by TabPFN at inference time implicitly represents a Bayesian ensemble, jointly integrating over a weighted hyperparameter space. The specific hyperparameter ranges and sampling strategies are chosen to cover a diverse set of scenarios that we expect to encounter in real-world tabular data.

#### Graph structure sampling

The structural causal models underlying each dataset are based on a DAG ${\mathcal{G}}$. We sample these graphs using the growing network with redirection sampling method^57^, a preferential attachment process that generates random scale-free networks. We either sample a single connected component or merge multiple disjoint subgraphs. Disjoint subgraphs lead to features that are marginally independent of the target if they are not connected to the target node, reflecting real-world scenarios with uninformative predictors.

To control the complexity of the sampled DAGs, we use two hyperparameters: the number of nodes *N* and the redirection probability *P*. *N* is sampled from a log-uniform distribution, $\log N \sim {\mathcal{U}}(a,b)$, where *a* and *b* are hyperparameters controlling the range of the graph size. The redirection probability *P* is sampled from a gamma distribution, *P* ~ *Γ*(*α*, *β*), where *α* and *β* are shape and rate parameters, respectively. Larger values of *N* yield graphs with more nodes, whereas smaller values of *P* lead to denser graphs with more edges on average^57^.

#### Computational edge mappings

In our implementation, each SCM node and sample is represented as a vector in ${{\mathbb{R}}}^{d}$. When propagating data through the SCM, the deterministic functions *f*_*i*_ at each edge map the input vectors to an output vector using four types of computational modules:

1. Small neural networks: here we initialize weight matrices $W\in {{\mathbb{R}}}^{d\times d}$ using Xavier initialization^58^ and apply a linear transformation *Wx* + *b* to the input vectors $x\in {{\mathbb{R}}}^{d}$, where $b\in {{\mathbb{R}}}^{d}$ is a bias vector. After the linear projection, we apply element-wise nonlinear activation functions $\sigma :{{\mathbb{R}}}^{d}\to {{\mathbb{R}}}^{d}$, randomly sampled from a set, including identity, logarithm, sigmoid, absolute value, sine, hyperbolic tangent, rank operation, squaring, power functions, smooth ReLU^59^, step function and modulo operation.
2. Categorical feature discretization: to generate categorical features from the numerical vectors at each node, we map the vector to the index of the nearest neighbour in a set of per node randomly sampled vectors {*p*_1_, …, *p*_*K*_} for a feature with *K* categories. This discrete index will be observed in the feature set as a categorical feature. We sample the number of categories *K* from a rounded gamma distribution with an offset of 2 to yield a minimum number of classes of 2. To further use these discrete class assignments in the computational graph, they need to be embedded as continuous values. We sample a second set of embedding vectors $\{{p}_{1}^{{\prime} },\ldots ,{p}_{K}^{{\prime} }\}$ for each class and transform the classes to these embeddings.
3. Decision trees: to incorporate structured, rule-based dependencies, we implement decision trees in the SCMs. At certain edges, we select a subset of features and apply decision boundaries on their values to determine the output^60^. The decision tree parameters (feature splits, thresholds) are randomly sampled per edge.
4. Noise injection: at each edge, we add random normal noise from the normal distribution ${\mathcal{N}}(0,{\sigma }^{2}I)$.

#### Initialization data sampling

For each to-be-generated sample, we randomly generate initialization data *ϵ* that is inserted at the DAG root nodes and then propagated through the computational graph. The noise variables *ϵ* are generated according to one of three sampling mechanisms:

1. Normal: ${\epsilon } \sim {\mathcal{N}}(0,{\sigma }_{{\epsilon }}^{2})$, where ${\sigma }_{{\epsilon }}^{2}$ is a hyperparameter.
2. Uniform: ${\epsilon } \sim {\mathcal{U}}(-a,a)$, where *a* is a hyperparameter.
3. Mixed: for each root node, we randomly select either a normal or uniform distribution to sample the initialization noise *ϵ* from.

Furthermore, we sample input data with varying degrees of non-independence for some datasets. Here we first sample a random fraction *ρ* of samples to serve as prototypes ${x}_{1}^{* },\ldots ,{x}_{M}^{* }$, where *M* = *ρn* and *n* is the dataset size. Then, for each input vector *x*_*i*_ to be sampled, we assign weights *α*_*ij*_ to the prototypes and linearly mix the final input as

1 $${x}_{i}=\mathop{\sum }\limits_{j=1}^{M}{\alpha }_{ij}{x}_{j}^{* },$$

where ∑_*j*_*α*_*ij*_ = 1. The weights *α*_*ij*_ are sampled from a multinomial distribution, *α*_*i*_ ~ Multinomial(*β*), where *β* is a temperature hyperparameter controlling the degree of non-independence: larger *β* yields more uniform weights, whereas smaller *β* concentrates the weights on fewer prototypes per sample.

#### Post-processing

Each dataset is post-processed randomly with one or more of the following post-processings: (1) For some datasets, we use the Kumaraswamy feature warping, introducing nonlinear distortions^33^ to features as done in ref. ^61^. (2) We quantize some continuous features into buckets of randomly sampled cardinality *K*, mimicking binned or discretized features commonly encountered in datasets. We map a feature value *x* to the index of the bucket it falls into, determined by *K* + 1 bin edges sampled from the set of values this feature takes. (3) To introduce scenarios for dynamic imputation and handling of incomplete datasets, a common challenge in data science, we randomly designate a fraction *ρ*_miss_ of the data as missing according to the missing completely at random strategy. Each value is masked as missing with probability *ρ*_miss_, independently of the data values.

#### Target generation

To generate target labels for regression tasks, we select a randomly chosen continuous feature without post-processing. For classification labels, we select a random categorical feature that contains up to 10 classes. Thus, natively our method is limited to predicting at most 10 classes. This number can be increased by pre-training on datasets with a larger number of classes or by using approaches such as building a one-vs-one classifier, one-vs-rest classifier or building on approaches such as error-correcting output codes (ECOC)^62^.

### Training details

The training loss of any PFN is the cross-entropy between the targets of held-out samples of synthetic datasets and the model prediction. For a test set (**X**_test_, ***y***_test_) = *D*_test_, the training loss is given by ${{\mathcal{L}}}_{{\rm{P}}{\rm{F}}{\rm{N}}}={{\bf{E}}}_{(({{\boldsymbol{X}}}_{{\rm{t}}{\rm{e}}{\rm{s}}{\rm{t}}},{{\boldsymbol{y}}}_{{\rm{t}}{\rm{e}}{\rm{s}}{\rm{t}}})\cup {D}_{{\rm{t}}{\rm{r}}{\rm{a}}{\rm{i}}{\rm{n}}})\sim p(D)}[-\log {q}_{\theta }({{\boldsymbol{y}}}_{{\rm{t}}{\rm{e}}{\rm{s}}{\rm{t}}}|{{\boldsymbol{X}}}_{{\rm{t}}{\rm{e}}{\rm{s}}{\rm{t}}},{D}_{{\rm{t}}{\rm{r}}{\rm{a}}{\rm{i}}{\rm{n}}})]$. By minimizing this loss, the PFN learns to approximate the true Bayesian posterior predictive distribution for a chosen prior over datasets (and potentially their latent variables) *D*, as shown in ref. ^22^.

We trained our final models for approximately 2,000,000 steps with a batch size of 64 datasets. That means the models used for TabPFN are trained on around 130,000,000 synthetically generated datasets each. One training run requires around 2 weeks on one node with eight Nvidia RTX 2080 Ti GPUs. We sample the number of training samples for each dataset uniformly up to 2,048 and use a fixed validation set size of 128. We sample the number of features using a beta distribution (*k* = 0.95, *b* = 8.0) that we linearly scale to the range 1–160. To avoid peaks in memory usage, the total size of each table was restricted to be below 75,000 cells by decreasing the number of samples for large numbers of features.

We chose the hyperparameters for the prior based on random searches, in which we use only a single GPU per training and evaluate on our development set, see section ‘Quantitative analysis’. We used the Adam optimizer^24^ with linear warmup and cosine annealing^63^ and tested a set of learning rates in [0.0001, 0.0005], using the one with the lowest final training loss.

### Inference details

To get the most performance out of TabPFN, it is crucial to optimize its inference pipeline. We generally always apply TabPFN in a small ensemble, in which we perform pre-processing or post-processing of the data differently for each ensemble member.

As our models are not fully permutation invariant, for each ensemble member, we shuffle the feature order, approximating order invariance^64^. For classification tasks, we additionally randomly permute the labels. We also apply a temperature to the softmax distribution of our model outputs for calibration.

Apart from the above, we use a subset of the following for each of our default ensemble members:

1. Quantile + Id: we quantize the inputs to equally spaced values between 0 and 1, but keep a copy of each original feature. This effectively doubles the number of features passed to TabPFN.
2. Category shuffling: the labels of categorical features with low cardinality are shuffled.
3. SVD: an SVD compression of the features is appended to the features.
4. Outlier removal: all outliers, more than 12 standard deviations from the mean, are removed.
5. Power transform: each feature (or the label for regression) is transformed using a Yeo–Johnson transformation to stabilize the variance and make the data more normally distributed.
6. One-hot encoding: categorical features are encoded using one-hot encoding, in which each category is represented as a binary vector.

For PHE and hyperparameter tuning of TabPFN, we use a larger set of pre-processing techniques that additionally include a logarithmic, an exponential and a KDI transformation^65^. These transformations help address nonlinear relationships, skewed distributions and varying scales among features.

To calibrate prediction uncertainty, we apply a softmax temperature (default *T* = 0.9) by dividing logits before the softmax calculation:

2 $$P({y}_{i}| x)=\frac{\exp ({z}_{i}/T)}{{\sum }_{j}\exp ({z}_{j}/T)},$$

where *z*_*i*_ are the logits, *T* is the temperature and *P*(*y*_*i*_∣*x*) is the calibrated probability. We offer the option to generate second-order polynomial features by multiplying up to 50 randomly selected feature pairs:

3 $${f}_{ij}={x}_{i}\cdot {x}_{j},\quad \,{\rm{for}}\,(i,j)\in {\mathcal{S}},$$

where ${\mathcal{S}}$ is the set of randomly chosen feature pairs. This can capture nonlinear interactions between features. This option is disabled by default. To ensure proper handling of duplicate samples given the sample permutation invariance of our architecture, we add a unique sample identifier feature. This is a random number drawn from a standard normal distribution, ensuring each sample is treated distinctly in the attention mechanism. We also provide an option for subsampling in each estimator, to increase ensemble diversity, which performs random sampling without replacement. This option is disabled by default.

#### Regression details

To enable our model to do classification on a large range of scales and target distributions, we use the following approach. During pre-training, we rescale our regression targets to have zero mean and a standard deviation of 1 (*z*-score). To decide where the borders between our features lie, we draw a large sample of datasets from our prior and choose the 1/5,000 quantiles from this distribution. At inference time, we bring the real-world data to a similar range by again applying *z*-score normalization. Furthermore, we allow applying a range of transforms, including a power transform as part of our default. All of the transforms, including the *z*-score are inverted at prediction time by applying the inverse of the transform to the borders between buckets. This is equivalent to applying the inverse of the transform to the random variable represented by our output distribution but for the half-normals used on the sides for full support^22^. This is because all transforms are strictly monotone and the borders represent positions on the cumulative distribution function.

#### Data grouping based on random forest

To perform well on very heterogeneous datasets, we also propose to use random trees to split the training data into smaller more homogeneous datasets. This technique is used only when performing HPO or PHE for TabPFN. It is especially useful for TabPFN as our model performs best on small datasets.

The pre-processing for a single ensemble member, that is, a single tree, works as follows: we use a standard random tree with feature and sample bootstrapping and Gini impurity loss. For each leaf node of the decision tree, we store the subset of training samples that fall into that node and train a TabPFN on these. To predict the class label for a test sample *x*, we determine the TabPFN to use by passing *x* through the decision tree. We set the minimal leaf size to be large (500–2,000) such that the resulting data groups are large enough to train a strong model.

### TabPFN (PHE)

To further enhance the inference performance of TabPFN, in TabPFN (PHE), we use PHE for a fixed portfolio of TabPFN configurations from our search space detailed in Extended Data Table 5. For TabPFN (PHE), we first use holdout validation to sequentially evaluate models from the portfolio until a time limit is reached. After all models are evaluated once, we repeat holdout validation with new data splits until the time limit is reached. Then, we ensemble all evaluated TabPFN models by aggregating their predictions with a weighted arithmetic mean. We learn the weights using greedy ensemble selection (GES)^42,66^ with 25 iterations on prediction data from holdout validation. Finally, we prune each zero-weighted model, refit all remaining models on all data and return the weighted average of their predictions.

Following standard practice in AutoML, we use GES because its predictive performance is often superior to the best individual model^43,67–69^. Owing to its ICL, we expect TabPFN to overfit the training data less than predictions of traditionally trained algorithms; thus, we opt for (repeated) holdout validation (as in Auto-Sklearn 1; ref. ^67^) instead of (repeated) cross-validation (as in AutoGluon^40^). Moreover, as GES usually produces sparse weight vectors^43,69^, we expect the final ensemble after pruning each zero-weighted model to consist of a smaller number of models than for other ensembling approaches, such as bagging. Consequently, PHE can also improve the inference efficiency of a TabPFN ensemble compared with other ensembling approaches.

### Foundation model abilities

#### Density estimation

The combination of a regression and a classification TabPFN can be used as a generative model for tabular data, not only modelling targets but features as well. Let ${\mathcal{D}}={\{({{\bf{x}}}_{i},{y}_{i})\}}_{i=1}^{N}$ denote the original dataset, where ${{\bf{x}}}_{i}\in {{\mathbb{R}}}^{d}$ is a *d*-dimensional feature vector and *y*_*i*_ is the corresponding target value, and let *q*_*θ*_ represent our trained TabPFN model, either a regression or classification model depending on the target type. We aim to approximate the joint distribution of a new example and its label $p({\bf{x}},y| {\mathcal{D}})$. To do this, we factorize the joint distribution as

4 $$p({\bf{x}},y| {\mathcal{D}})=\mathop{\prod }\limits_{j=1}^{d}p({x}_{j}| {{\bf{x}}}_{ < j},{\mathcal{D}})\cdot p(\,y| {\bf{x}},{\mathcal{D}})$$

5 $$\approx \mathop{\prod }\limits_{j=1}^{d}{q}_{\theta }({x}_{j}| {{\boldsymbol{x}}}_{ < j},{{\mathcal{D}}}_{:, < j})\cdot {q}_{\theta }(\,y| {\boldsymbol{x}},{\mathcal{D}}),$$

where we only condition on a subset of the features in the training set (${{\mathcal{D}}}_{:, < j}$). The feature order of the joint density factorization influences the estimated densities. To reduce variance from this source, we apply a permutation sampling approximation of Janossy Pooling at inference time, in which we average the outputs of *N*_*j*_ feature permutations, with *N*_*j*_ = 24 in our experiments^64^.

As we cannot condition on an empty feature set for technical reasons, we condition the prediction of the first feature *x*_1_, on a feature with random noise, that is, no information.

The above factorization of the density of a sample (equation (5)) is completely tractable and we thus use it to estimate the likelihood for data points. This enables tasks such as anomaly detection and outlier identification.

#### Synthetic data generation

We can leverage the generative abilities of TabPFN (see section ‘Density estimation’) to synthesize new tabular data samples that mimic the characteristics of a given real-world dataset, by simply following the factorization in equation (5) and sampling each feature step by step. The generated synthetic samples (x^*^, y^*^) can be used for various purposes, such as data augmentation, privacy-preserving data sharing and scenario simulation.

#### Embeddings

TabPFN can be used to retrieve meaningful feature representations or embeddings. Given a dataset ${\mathcal{D}}={\{({{\bf{x}}}_{i},{y}_{i})\}}_{i=1}^{N}$, the goal is to learn a mapping ${f}_{\theta }:{{\mathbb{R}}}^{d}\to {{\mathbb{R}}}^{k}$ that transforms the original *d*-dimensional feature vectors **x**_*i*_ into an embedding space of dimension *k*. The resulting embeddings ${f}_{\theta }({{\bf{x}}}_{i})\in {{\mathbb{R}}}^{k}$ capture the learned relationships between features and can be used for downstream tasks. To use TabPFN for this problem, we simply use the target-column representations of its final layer as embeddings.

### Detailed evaluation protocol

To rigorously assess the performance and robustness of TabPFN, we conduct a comprehensive quantitative evaluation on standard tabular dataset benchmarks, comparing against state-of-the-art baselines under a standardized protocol.

#### Default configuration of TabPFN

Unlike traditional algorithms, in-context-learned algorithms do not have hyperparameters that directly control their training procedure. Instead, hyperparameters for inference of TabPFN only control the pre-processing of data and post-processing of predictions (for example, feature scaling or softmax temperature). Our default configuration (TabPFN (default)) for both classification and regression is optimized for accurate predictions with minimal fitting time. Here, we apply the same model multiple times with different pre- and post-processors and take the average over the predictions, yielding a four-way (eight-way for regression) ensemble. The settings for our data processing were obtained through a hyperparameter search optimized on our development datasets. The exact settings chosen are listed in Extended Data Table 5. We emphasize that, as for other foundation models (such as GPT), we trained our TabPFN model once and used the same model to perform ICL in a forward pass on all new datasets.

#### Baselines

We compare with tree-based methods, such as random forests^38^, XGBoost^7^, CatBoost^9^ and LightGBM^8^, the state of the art for experts to perform predictions on tabular data^14,15^. We also compare with simpler methods, such as ridge regression^70^, logistic regression and SVMs^39^. Although standard neural networks, which unlike TabPFN do not use ICL, were shown to underperform for small (<10,000 samples) tabular data^1,14,71^, as a point of reference, we still consider a simple neural network, the MLP.

#### Tabular dataset benchmarks

We perform our analysis on two widely used and publicly available benchmark suites: the standard AutoML benchmark^36^ and the recent regression benchmark OpenML-CTR23 (ref. ^37^). Both benchmarks comprise a diverse set of real-world tabular datasets, carefully curated to be representative of various domains and data characteristics. The authors of the benchmark suite selected these datasets based on criteria such as sufficient complexity, real-world relevance, absence of free-form text features and diversity of problem domains.

For our quantitative analysis of TabPFN for classification tasks, we use a set of test datasets comprising all 29 datasets from the AutoML benchmark with up to 10,000 samples, 500 features and 10 classes. For regression tasks, the AutoML benchmark contains only 16 datasets matching these constraints. To increase statistical power, we augmented this set with all datasets matching our constraints from the recent OpenML-CTR23 benchmark, yielding a test set of 28 unique regression datasets in total. Extended Data Tables 3 and 4 provide full details for our test sets of classification and regression datasets, respectively.

We further evaluated additional benchmark suites from refs. ^14,15^. In ref. ^14^, there are 22 tabular classification datasets selected based on criteria such as heterogeneous columns, moderate dimensionality and sufficient difficulty. In ref. ^15^, there is a collection of 176 classification datasets, representing one of the largest tabular data benchmarks. However, the curation process for these datasets may not be as rigorous or quality controlled as for AutoML Benchmark and OpenML-CTR23. We also evaluated five Kaggle competitions with less than 10,000 training samples from the latest completed Tabular Playground Series.

#### Development datasets

To decide on the hyperparameters of TabPFN, as well as our hyperparameter search spaces, we considered another set of datasets, our development datasets. We carefully selected datasets to be non-overlapping with our test datasets described above. The list of development datasets can be found in Supplementary Tables 5 and 6. We considered the mean of normalized scores (ROC/RMSE) and rank quantiles and chose the best model configurations on these development datasets.

#### Metrics and cross-validation

To obtain scores for classification tasks, we use two widely adopted evaluation metrics: ROC AUC (One-vs-Rest) and accuracy. ROC AUC averages performance over different sensitivity–specificity trade-offs, and accuracy measures the fraction of samples labelled correctly.

For regression tasks, we use *R*^2^ and negative RMSE as evaluation metrics. *R*^2^ represents the proportion of variance in the target column that the model can predict. RMSE is the root of the average squared magnitude of the errors between the predicted and actual values. As we use negative RMSE, for all our four metrics higher values indicate a better fit.

To increase statistical validity, for each dataset and method in our test datasets, we evaluated 10 repetitions, each with a different random seed and train–test split (90% train and 10% test samples; all methods used the same cross-validation splits, defined by OpenML^72^). We average the scores of all repetitions per dataset. Then, to average scores across datasets, we normalize per dataset following previous benchmarks^36,40^. The absolute scores are linearly scaled such that a score of 1.0 corresponds to the highest value achieved by any method on that dataset, whereas a score of 0 represents the lowest result. This normalization allows for building meaningful averages across datasets with very different score ranges. We provide absolute performance numbers in Supplementary Data Tables 1–2. All confidence intervals shown are 95% confidence intervals.

**Extended Data Fig. 2:**
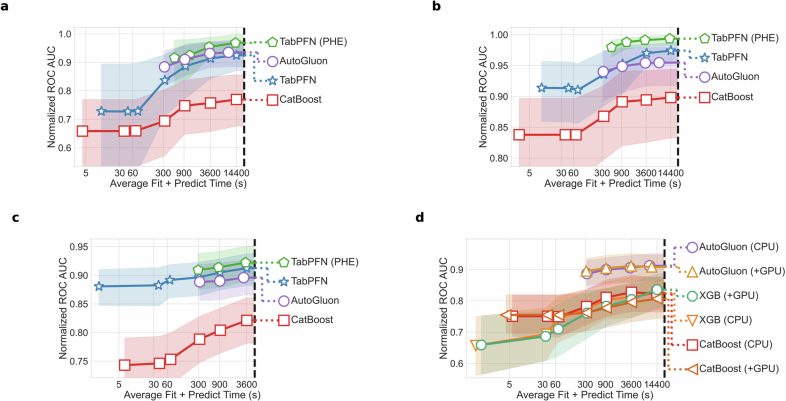
Performance comparisons of TabPFN and baselines on additional benchmark datasets and with GPU support. (a) Classification performance on the Grinsztajn medium-sized benchmark with categorical features, across 7 datasets. (b) Classification performance on the Grinsztajn medium-sized benchmark with numerical features, across its 15 datasets. (c) Classification performance on the TabZilla benchmark, consisting of 102 datasets with fewer than 10,000 rows of data, 500 features, and 10 classes. Duplicated datasets and those with fewer than 5 samples per class were removed to enable 5-fold cross-validation. (d) Performance Over Time Comparison with CPU vs. GPU Hardware: The performance over time when running our strongest baselines with eight CPUs (CPU) vs. eight CPUs and on one GPU (+GPU) on our classification test benchmark. AutoGluon automatically decides which models to train with what resources. For CatBoost and XGB, we specified that the models should train with GPU. Intervals represent 95% CI.

**Extended Data Fig. 3:**
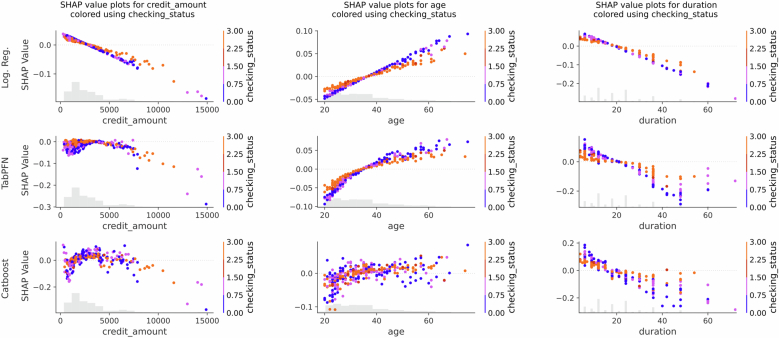
Comparing SHAP (SHapley Additive exPlanations) summary plots between TabPFN and baselines. We compare SHAP feature importance and impact for Logistic Regression, TabPFN, and CatBoost on the “Default of Credit Card Clients” dataset. The top features visualized are credit amount, age, and duration. Each point represents a single instance, with the color indicating the value of the checking status feature (blue for low, red for high), illustrating its interaction with the respective feature on the x-axis. We see that Logistic Regression is most interpretable due to the simple underlying functions. However, Logistic Regression has poor predictive accuracy, and the learned functions are unintuitive when looking at the outer bounds of features. TabPFN has good predictive accuracy and learns simple, interpretable functions. CatBoost is the least interpretable, with unclear patterns and wide variation in SHAP values per sample. This figure is adapted from Lundberg et al.^47^.

**Extended Data Fig. 4:**
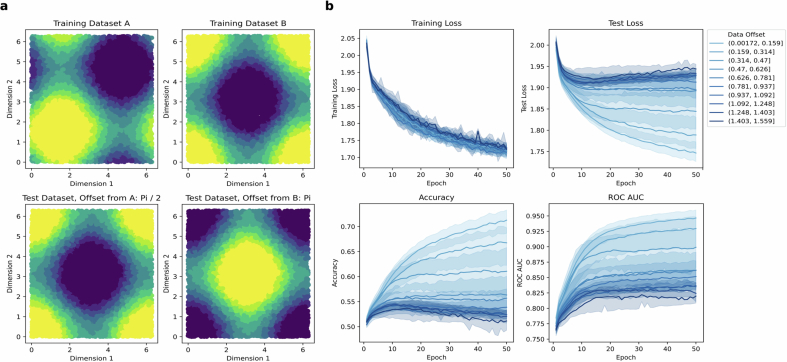
Finetuning TabPFN on 2-dimensional sine curve datasets. (a) Examples of 2D sine curve datasets with different offsets. (b) Finetuning loss curves for 50 runs with random train-test offsets. Colors indicate the offset between train and test. TabPFN shows positive transfer, with better performance for more similar distributions. For a dataset shift of *π*, the inverse label needs to be predicted in the test set, compared to the finetuning data. However, TabPFN still generalizes when finetuned on this data.

We tuned all methods with a random search using five-fold cross-validation with ROC AUC/RMSE up to a given time budget, ranging from half a minute to 4 h. The first candidate in the random search was the default setting supplied in the implementation of the method and was also used if not a single cross-validation run finished before the time budget was consumed. See the section ‘Qualitative analysis’ for the used search spaces per method. All methods were evaluated using 8 CPU cores. Moreover, TabPFN makes use of a 5-year-old consumer-grade GPU (RTX 2080 Ti). We also tested GPU acceleration for the baselines. However, as Extended Data Fig. 2 shows, this did not improve performance, probably because of the small dataset sizes.

## Online content

Any methods, additional references, Nature Portfolio reporting summaries, source data, extended data, supplementary information, acknowledgements, peer review information; details of author contributions and competing interests; and statements of data and code availability are available at 10.1038/s41586-024-08328-6.

## Supplementary information

Supplementary Tables 1–4 Unnormalized per dataset results: per dataset ROC AUC scores for our model and baselines on the four evaluated benchmarks.

Supplementary Tables 5 and 6 Meta-information on development datasets: meta-information of the development dataset is used to validate the performance of our models for regression and classification.

## Figures and Tables

**Extended Data Table 1:**
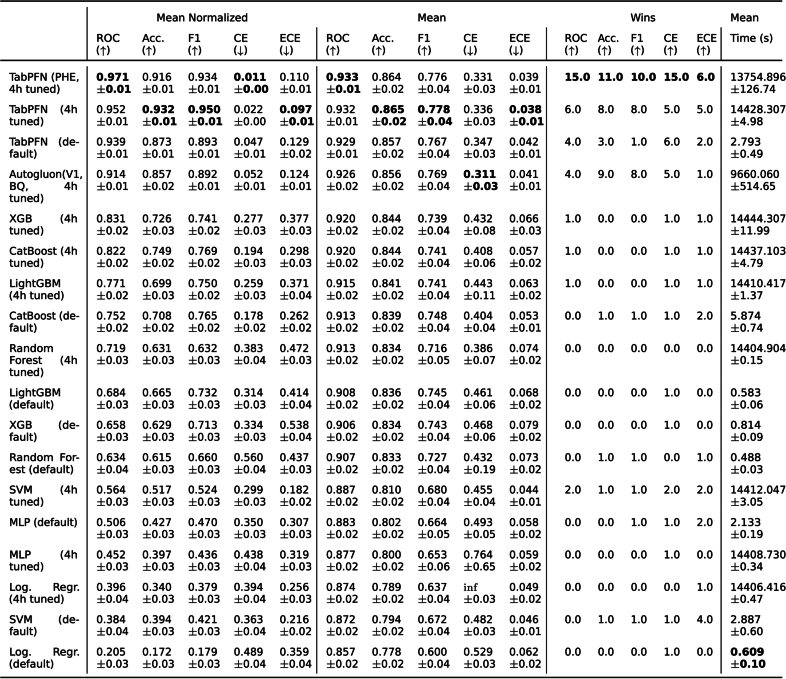
Aggregated results on the 29 AMLB classification Benchmark datasets

Scores are normalized on all the baselines shown in this table, with the weakest score set to 0.0 and the highest to 1.0, per dataset. All baselines are optimized for ROC AUC thus trading-off representativeness of secondary metrics. Times for TabPFN refer to times on GPU. Datasets are available via OpenML https://www.openml.org/search?type=data&sort=runs&id={OPENML_ID}. Exact train-test splits defined by OpenML tasks with task numbers in our code datasets/benchmark_dids.py.

**Extended Data Table 2:**
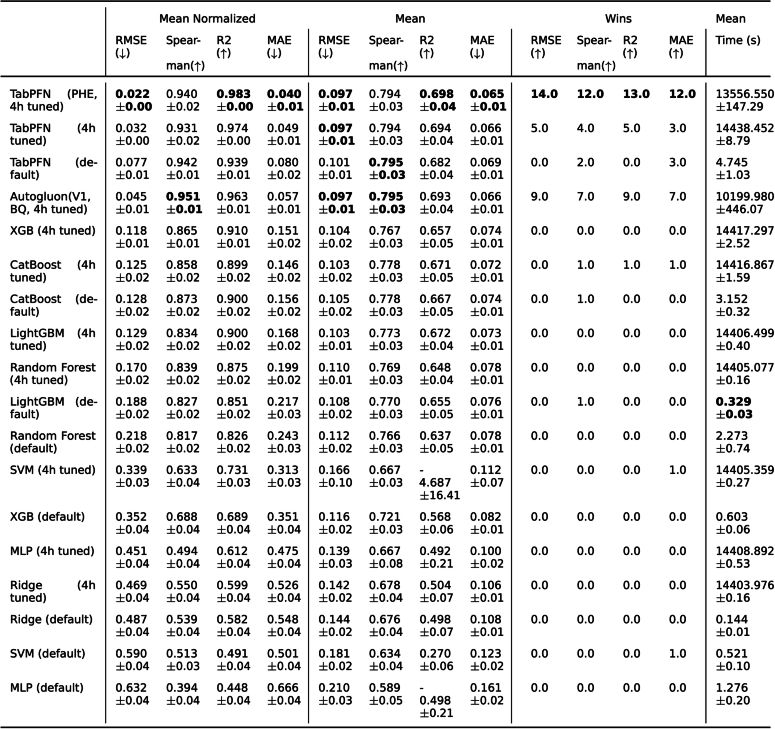
Aggregated results on the 28 AMLB and OpenML-CTR23 regression Benchmark datasets

Scores are normalized on all the baselines shown in this table, with the weakest score set to 0.0 and the highest to 1.0, per dataset. K-Nearest Neighbors^73^ performed significantly worse than the considered baselines. All baselines are optimized for RMSE as an objective thus trading-off representativeness of secondary metrics. Times for TabPFN refer to times on GPU. Datasets are available via OpenML https://www.openml.org/search?type=data&sort=runs&id={OPENML_ID}. Exact train-test splits defined by OpenML tasks with task numbers in our code datasets/benchmark_dids.py.

**Extended Data Table 3:**
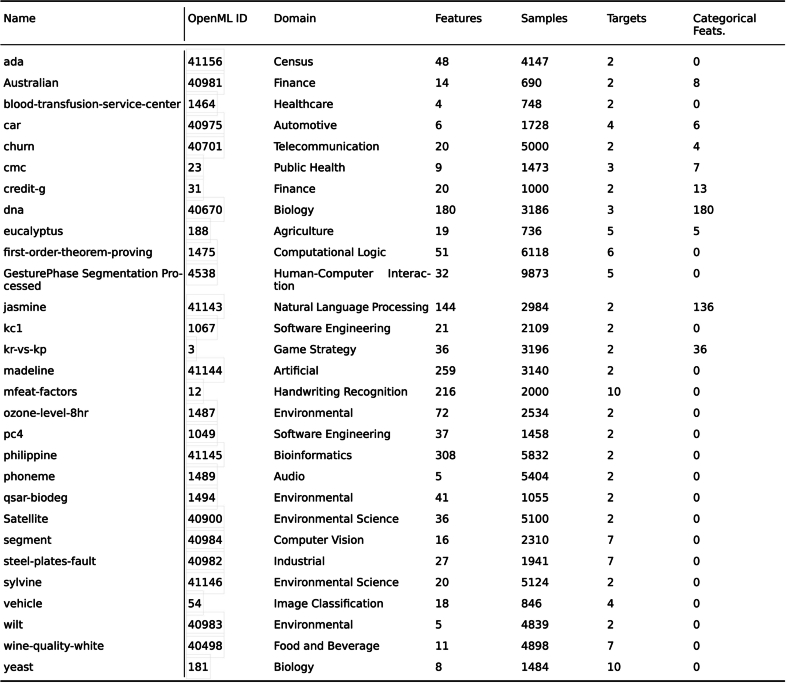
List of test datasets used for primary evaluation of classification tasks

All classification tasks from the AutoML Benchmark^36^ with fewer 10,000 samples and 500 features. The benchmark comprises diverse real-world tabular datasets, curated for complexity, relevance, and domain diversity.

**Extended Data Table 4:**
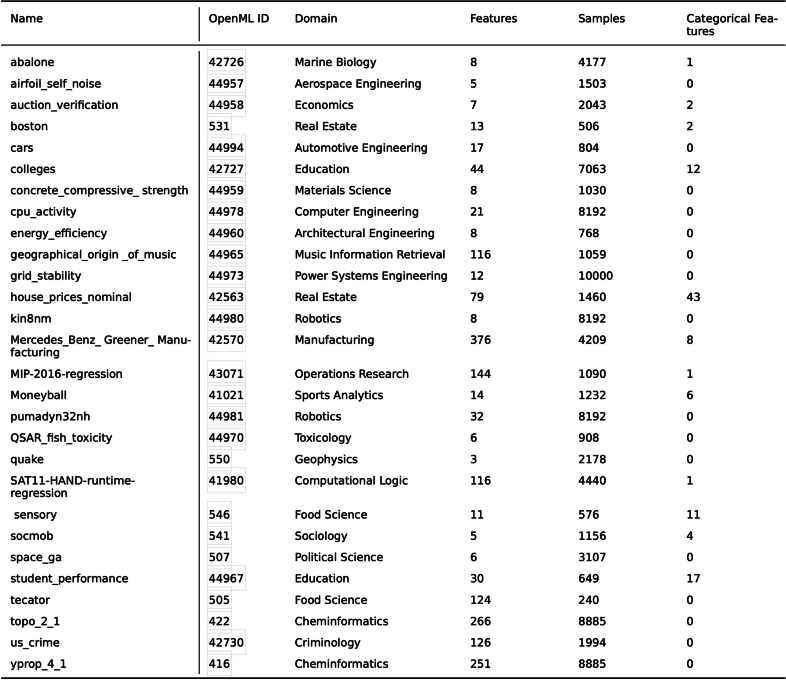
List of test datasets used for primary evaluation of regression tasks

All regression tasks from the AutoML^36^ and OpenML-CTR23^37^ Benchmarks with fewer 10,000 samples and 500 features. The benchmark comprises diverse real-world tabular datasets, curated for complexity, relevance, and domain diversity.

**Extended Data Table 5:**
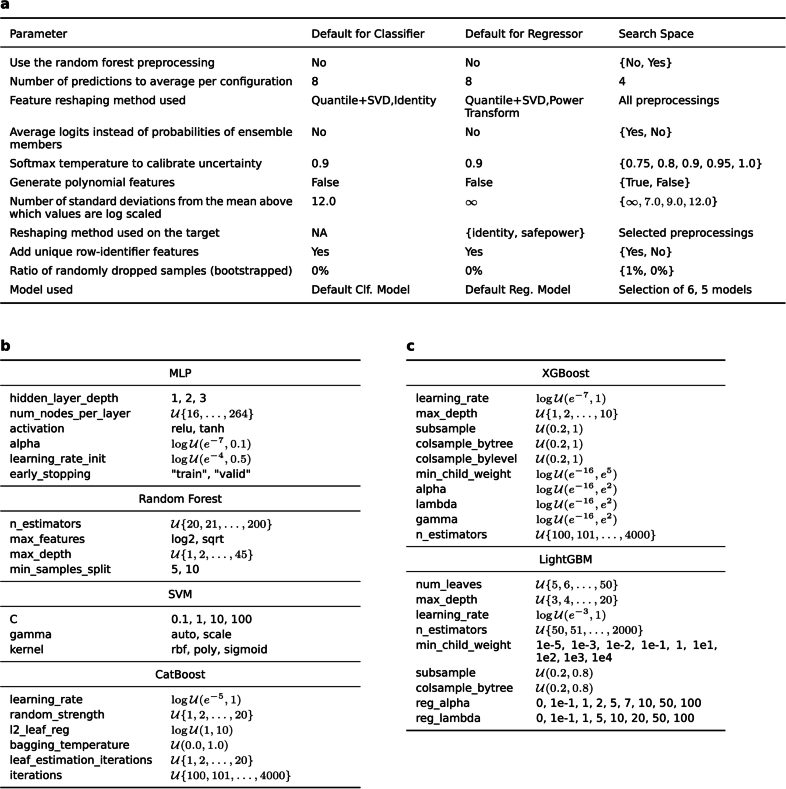
Hyperparameter defaults and search space for TabPFN and our baselines

(a) TabPFN search space (b, c) Baseline search spaces.

**Extended Data Table 6:**
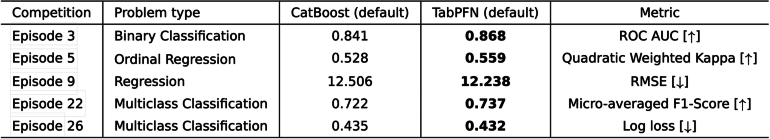
Performance on Kaggle Data Science Challenges

Performance of default CatBoost and default TabPFN on all 5 Kaggle classification or regression competitions from the Tabular Playground Series Season 3 with late submission enabled, fewer than 10,000 rows of data, 500 features, and 10 classes. We report the private score averaged over 5 seeds. For Episode 5, as ordinal regression can be treated as a classification or regression task, for both CatBoost and TabPFN we tried both the regression and the classification model and chose the better of the two (regression for CatBoost; classification for TabPFN). Arrows indicate the optimization direction for each metric. We emphasize that these results only compare Catboost and TabPFN on the raw competition data, not using any of the tricks the ingenious Kaggle community applies, such as use of domain knowledge, data cleaning, special feature engineering, postprocessing and ensembling; nevertheless, these techniques can be combined with TabPFN, and we hope that TabPFN’s improved base model performance will allow Kagglers to achieve even better results with them.

## Data Availability

All datasets evaluated are publicly available on openml.org or kaggle.com. We have provided scripts in our code repository that automate the process of downloading and evaluating the datasets. These scripts contain dataset identifiers, as well as exact data splitting and processing procedures.

## Code availability

Our code is available at https://priorlabs.ai/tabpfn-nature/ (10.5281/zenodo.13981285). We also provide an API that allows users to run TabPFN with minimal coding experience or without the availability of specific computing hardware such as a GPU. The code is designed to be modular and easily installable in a standard Python environment. The code to generate synthetic pre-training data has not been released with our models. We aim to enable researchers and practitioners to easily integrate TabPFN into their workflows and apply it to their specific tabular data tasks. We encourage users to provide feedback, report issues, and contribute to the further development of TabPFN. This open release aims to facilitate collaboration and accelerate the adoption and advancement of TabPFN in various research and application domains.
