# Supplementary material for: Accurate predictions on small data with a tabular foundation model
Source: Nature. 2025 Jan 8;637(8045):319–26. doi: 10.1038/s41586-024-08328-6 (PMC11711098; doi:10.1038/s41586-024-08328-6)
Supplement: Supplementary file 1 — Unnormalized per dataset results: per dataset ROC AUC scores for our model and baselines on the four evaluated benchmarks. [file 41586_2024_8328_MOESM1_ESM.pdf]

1 Supplementary Tables

Supplementary Table 1: **Raw Performance Scores per Dataset for the Classification Test Benchmark.** The table presents the mean and standard deviation of the ROC AUC score across all folds per dataset for each compared method after four hours. These are the raw results used to generate our main classification results. The second column details the OpenML tasks and dataset IDs.

Dataset Name	Task (Dataset)	AutoGluon	CatBoost	KNN	LightGBM	RF	Logistic Regression	MLP	SVM	XGB	TabPFN (PHE)	TabPFN
pc4	359958 (1049)	0.9534 (± 0.0210)	0.9511 (± 0.0179)	0.8439 (± 0.0505)	0.9429 (± 0.0229)	0.9453 (± 0.0183)	0.9065 (± 0.0307)	0.9234 (± 0.0246)	0.9053 (± 0.0361)	0.9493 (± 0.0186)	**0.9577 (± 0.0165)**	0.9569 (± 0.0169)
kc1	359962 (1067)	0.8418 (± 0.0311)	0.8255 (± 0.0399)	0.7619 (± 0.0509)	0.8079 (± 0.0367)	0.8316 (± 0.0332)	0.7988 (± 0.0358)	0.8054 (± 0.0336)	0.7985 (± 0.0347)	0.8274 (± 0.0342)	**0.8519 (± 0.0279)**	0.8477 (± 0.0323)
mfeatfactors	359961 (12)	0.9996 (± 0.0005)	0.9989 (± 0.0015)	0.9969 (± 0.0027)	0.9991 (± 0.0011)	0.9986 (± 0.0015)	0.9985 (± 0.0016)	0.9989 (± 0.0014)	0.9995 (± 0.0007)	0.9991 (± 0.0011)	0.9997 (± 0.0003)	**0.9998 (± 0.0003)**
bloodtransfusionservicecenter	359955 (1464)	0.7590 (± 0.0456)	0.7565 (± 0.0543)	0.7292 (± 0.0595)	0.7440 (± 0.0496)	0.7346 (± 0.0513)	0.7592 (± 0.0435)	0.6790 (± 0.1092)	0.7364 (± 0.0501)	0.7528 (± 0.0487)	**0.7599 (± 0.0454)**	0.7575 (± 0.0411)
firstordertheoremproving	359969 (1475)	0.8607 (± 0.0088)	0.8519 (± 0.0088)	0.7942 (± 0.0115)	0.8525 (± 0.0097)	0.8519 (± 0.0094)	0.7097 (± 0.0129)	0.7781 (± 0.0100)	0.7607 (± 0.0270)	0.8571 (± 0.0103)	**0.8618 (± 0.0088)**	0.8587 (± 0.0073)
ozonelevel8hr	190137 (1487)	0.9317 (± 0.0190)	0.9293 (± 0.0195)	0.8691 (± 0.0508)	0.9217 (± 0.0169)	0.9120 (± 0.0346)	0.9152 (± 0.0289)	0.9176 (± 0.0269)	0.9212 (± 0.0264)	0.9321 (± 0.0179)	**0.9343 (± 0.0179)**	0.9271 (± 0.0188)
phoneme	168350 (1489)	0.9733 (± 0.0075)	0.9640 (± 0.0092)	0.9319 (± 0.0137)	0.9599 (± 0.0098)	0.9631 (± 0.0099)	0.8136 (± 0.0219)	0.8913 (± 0.0109)	0.9127 (± 0.0153)	0.9667 (± 0.0081)	**0.9734 (± 0.0073)**	0.9729 (± 0.0076)
qsarbiodeg	359956 (1494)	**0.9421 (± 0.0322)**	0.9283 (± 0.0369)	0.9056 (± 0.0344)	0.9308 (± 0.0355)	0.9283 (± 0.0324)	0.9247 (± 0.0317)	0.9270 (± 0.0313)	0.9225 (± 0.0357)	0.9324 (± 0.0410)	0.9401 (± 0.0323)	0.9361 (± 0.0326)
yeast	2073 (181)	0.8638 (± 0.0352)	0.8796 (± 0.0325)	0.8441 (± 0.0252)	0.8514 (± 0.0384)	**0.8866 (± 0.0342)**	0.8616 (± 0.0400)	0.8651 (± 0.0410)	0.8673 (± 0.0472)	0.8762 (± 0.0303)	0.8822 (± 0.0431)	0.8819 (± 0.0443)
eucalyptus	359954 (188)	0.9328 (± 0.0170)	0.9159 (± 0.0191)	0.8439 (± 0.0153)	0.9130 (± 0.0180)	0.9142 (± 0.0183)	0.9138 (± 0.0178)	0.9095 (± 0.0153)	0.9067 (± 0.0128)	0.9049 (± 0.0231)	**0.9361 (± 0.0151)**	0.9341 (± 0.0149)
cmc	359959 (23)	0.7449 (± 0.0300)	0.7480 (± 0.0296)	0.6377 (± 0.0313)	0.7464 (± 0.0386)	0.7450 (± 0.0331)	0.7026 (± 0.0407)	0.6970 (± 0.0319)	0.6850 (± 0.0399)	0.7391 (± 0.0402)	0.7552 (± 0.0313)	**0.7568 (± 0.0284)**
creditg	168757 (31)	0.7968 (± 0.0338)	0.7972 (± 0.0317)	0.7431 (± 0.0298)	0.7873 (± 0.0491)	0.7998 (± 0.0327)	0.7879 (± 0.0410)	0.6698 (± 0.1041)	0.7855 (± 0.0205)	0.7962 (± 0.0367)	0.7966 (± 0.0356)	**0.8029 (± 0.0363)**
krvskp	359965 (3)	0.9998 (± 0.0003)	0.9997 (± 0.0004)	0.9900 (± 0.0040)	0.9997 (± 0.0005)	0.9987 (± 0.0016)	0.9955 (± 0.0028)	0.9996 (± 0.0004)	**0.9999 (± 0.0002)**	0.9995 (± 0.0009)	0.9995 (± 0.0006)	0.9997 (± 0.0005)
winequalitywhite	359974 (40498)	0.8629 (± 0.0334)	0.8613 (± 0.0194)	0.7094 (± 0.0208)	0.8518 (± 0.0292)	0.8468 (± 0.0284)	0.7226 (± 0.0468)	0.7508 (± 0.0419)	0.8007 (± 0.0334)	0.8674 (± 0.0301)	0.9035 (± 0.0171)	**0.9040 (± 0.0122)**
dna	359964 (40670)	0.9956 (± 0.0025)	0.9952 (± 0.0027)	0.9642 (± 0.0067)	0.9953 (± 0.0019)	0.9935 (± 0.0034)	0.9945 (± 0.0033)	0.9915 (± 0.0035)	0.9942 (± 0.0035)	0.9952 (± 0.0025)	**0.9956 (± 0.0023)**	0.9954 (± 0.0024)
churn	359968 (40701)	**0.9327 (± 0.0216)**	0.9224 (± 0.0254)	0.8479 (± 0.0335)	0.9198 (± 0.0277)	0.9166 (± 0.0210)	0.8621 (± 0.0208)	0.9129 (± 0.0194)	0.9013 (± 0.0225)	0.9203 (± 0.0294)	0.9210 (± 0.0155)	0.9298 (± 0.0174)
Satellite	359975 (40900)	0.9961 (± 0.0036)	0.9923 (± 0.0081)	0.9275 (± 0.0581)	0.9849 (± 0.0236)	0.9828 (± 0.0257)	0.9922 (± 0.0116)	0.9944 (± 0.0059)	0.9917 (± 0.0117)	0.9872 (± 0.0164)	**0.9977 (± 0.0014)**	0.9963 (± 0.0048)
car	359960 (40975)	**1.0000 (± 0.0000)**	**1.0000 (± 0.0000)**	0.9927 (± 0.0032)	**1.0000 (± 0.0000)**	0.9993 (± 0.0005)	0.9922 (± 0.0027)	0.9998 (± 0.0003)	**1.0000 (± 0.0000)**	1.0000 (± 0.0000)	**1.0000 (± 0.0000)**	**1.0000 (± 0.0000)**
Australian	146818 (40981)	0.9428 (± 0.0216)	0.9406 (± 0.0223)	0.9216 (± 0.0278)	0.9413 (± 0.0204)	0.9389 (± 0.0246)	0.9309 (± 0.0244)	0.9236 (± 0.0264)	0.9299 (± 0.0185)	0.9425 (± 0.0163)	**0.9456 (± 0.0203)**	0.9433 (± 0.0217)
steelplatesfault	168784 (40982)	0.9716 (± 0.0038)	0.9694 (± 0.0058)	0.9245 (± 0.0126)	0.9668 (± 0.0061)	0.9640 (± 0.0051)	0.9326 (± 0.0087)	0.9433 (± 0.0089)	0.9502 (± 0.0084)	0.9691 (± 0.0035)	**0.9788 (± 0.0035)**	0.9785 (± 0.0032)
wilt	146820 (40983)	**0.9955 (± 0.0065)**	0.9930 (± 0.0079)	0.8539 (± 0.0447)	0.9922 (± 0.0083)	0.9877 (± 0.0137)	0.9751 (± 0.0111)	0.9410 (± 0.0346)	0.9937 (± 0.0050)	0.9909 (± 0.0103)	0.9947 (± 0.0088)	0.9948 (± 0.0086)
segment	359963 (40984)	0.9968 (± 0.0012)	0.9961 (± 0.0017)	0.9867 (± 0.0037)	0.9956 (± 0.0022)	0.9956 (± 0.0014)	0.9853 (± 0.0047)	0.9851 (± 0.0046)	0.9916 (± 0.0033)	0.9963 (± 0.0015)	0.9972 (± 0.0012)	**0.9974 (± 0.0012)**
jasmine	168911 (41143)	0.8873 (± 0.0165)	0.8825 (± 0.0213)	0.8519 (± 0.0257)	0.8694 (± 0.0169)	0.8821 (± 0.0141)	0.8496 (± 0.0160)	0.8355 (± 0.0295)	0.8584 (± 0.0197)	0.8855 (± 0.0180)	**0.8913 (± 0.0140)**	0.8879 (± 0.0163)
madeline	190392 (41144)	0.9478 (± 0.0085)	0.9382 (± 0.0073)	0.6981 (± 0.0307)	0.9304 (± 0.0121)	0.8721 (± 0.0150)	0.6454 (± 0.0298)	0.7127 (± 0.0319)	0.7015 (± 0.0285)	0.9433 (± 0.0090)	**0.9750 (± 0.0048)**	0.9748 (± 0.0048)
philippine	190410 (41145)	0.8799 (± 0.0135)	0.8681 (± 0.0158)	0.7735 (± 0.0120)	0.8678 (± 0.0154)	0.8441 (± 0.0161)	0.8018 (± 0.0218)	0.8025 (± 0.0188)	0.7973 (± 0.0205)	0.8747 (± 0.0133)	**0.9751 (± 0.0050)**	0.9724 (± 0.0049)
sylvine	359972 (41146)	0.9926 (± 0.0035)	0.9885 (± 0.0044)	0.8931 (± 0.0096)	0.9885 (± 0.0044)	0.9822 (± 0.0046)	0.9660 (± 0.0063)	0.9745 (± 0.0057)	0.9731 (± 0.0065)	0.9882 (± 0.0032)	**0.9972 (± 0.0017)**	0.9964 (± 0.0025)
ada	190411 (41156)	0.9220 (± 0.0171)	0.9199 (± 0.0173)	0.8721 (± 0.0207)	0.9190 (± 0.0177)	0.9142 (± 0.0173)	0.9065 (± 0.0209)	0.8956 (± 0.0216)	0.8994 (± 0.0203)	**0.9230 (± 0.0188)**	0.9182 (± 0.0184)	0.9172 (± 0.0190)
GesturePhaseSegmentationProcessed	359970 (4538)	0.9448 (± 0.0070)	0.9200 (± 0.0074)	0.8380 (± 0.0108)	0.9168 (± 0.0070)	0.8978 (± 0.0083)	0.7617 (± 0.0090)	0.7825 (± 0.0110)	0.7941 (± 0.0041)	0.9210 (± 0.0073)	**0.9493 (± 0.0054)**	0.9437 (± 0.0047)
vehicle	190146 (54)	**0.9756 (± 0.0061)**	0.9376 (± 0.0137)	0.9031 (± 0.0122)	0.9322 (± 0.0105)	0.9315 (± 0.0125)	0.9486 (± 0.0121)	0.9340 (± 0.0196)	0.9664 (± 0.0101)	0.9400 (± 0.0106)	0.9738 (± 0.0052)	0.9732 (± 0.0068)

Supplementary Table 2: **Raw Performance Scores per Dataset for the Regression Test Benchmark.** The table presents the mean and standard deviation of the negative RMSE across all folds per dataset for each compared method after four hours. These are the raw results used to generate our main regression results. The second column details the OpenML tasks and dataset IDs.

Dataset Name	Task (Dataset)	AutoGluon	CatBoost	KNN	LightGBM	RF	Linear Regression	MLP	SVM	XGB	TabPFN (PHE)	TabPFN
Moneyball	167210 (41021)	-21.0201 (± 0.8042)	-21.8662 (± 0.9489)	-43.7007 (± 3.9935)	-22.2264 (± 1.3133)	-24.4470 (± 1.4166)	-21.1813 (± 0.7308)	-21.5401 (± 0.8635)	-22.4235 (± 1.0117)	-22.4115 (± 1.0351)	**-20.7921 (± 0.7502)**	-20.9331 (± 0.6911)
yprop_4_1	359940 (416)	**-0.0277 (± 0.0049)**	-0.0280 (± 0.0048)	-0.0287 (± 0.0047)	-0.0281 (± 0.0049)	-0.0278 (± 0.0049)	-0.0286 (± 0.0049)	-0.0328 (± 0.0121)	-0.1389 (± 0.3409)	-0.0278 (± 0.0049)	-0.0280 (± 0.0049)	-0.0281 (± 0.0049)
SAT11HANDruntimeregression	359948 (41980)	**-867.5137 (± 57.3159)**	-1087.0711 (± 55.5359)	-1441.9111 (± 73.3988)	-929.8491 (± 61.6588)	-1103.3545 (± 50.0841)	-1572.6944 (± 61.7066)	-1140.7448 (± 49.7484)	-1834.0961 (± 56.7694)	-916.4924 (± 59.0278)	-1000.6162 (± 70.5234)	-1032.4170 (± 53.9486)
topo_2_1	359939 (422)	**-0.0281 (± 0.0049)**	-0.0283 (± 0.0048)	-0.0293 (± 0.0048)	-0.0283 (± 0.0048)	-0.0285 (± 0.0048)	-0.0286 (± 0.0049)	-0.0296 (± 0.0048)	-0.0321 (± 0.0040)	-0.0282 (± 0.0048)	-0.0282 (± 0.0049)	-0.0284 (± 0.0049)
house_prices_nominal	359951 (42563)	-25931.8375 (± 9655.1239)	-24780.5884 (± 7621.4787)	-41721.4321 (± 7233.6608)	-26001.7092 (± 6138.2257)	-29216.3685 (± 6002.6551)	-33163.3526 (± 11029.7956)	-35394.7936 (± 10908.9774)	-72992.1840 (± 6886.7809)	-36408.5046 (± 7807.0299)	**-21826.5690 (± 6841.0495)**	-23394.6543 (± 9484.7589)
Mercedes_Benz_Greener_Manufacturing	233215 (42570)	-8.2764 (± 1.0832)	-8.2710 (± 1.1026)	-9.2702 (± 0.9814)	-8.3343 (± 1.0383)	-8.4401 (± 1.0373)	-8.5241 (± 1.0692)	-8.6180 (± 1.0508)	-8.6570 (± 1.0961)	-11.9073 (± 1.7739)	**-8.2135 (± 1.0933)**	-8.2414 (± 1.0800)
abalone	359944 (42726)	-2.0515 (± 0.1213)	-2.1367 (± 0.1159)	-2.1963 (± 0.1235)	-2.1284 (± 0.1201)	-2.1319 (± 0.1167)	-2.2122 (± 0.1383)	-2.1930 (± 0.1210)	-2.1295 (± 0.1263)	-2.1117 (± 0.1185)	**-2.0330 (± 0.1139)**	-2.0340 (± 0.1075)
colleges	359942 (42727)	**-0.1354 (± 0.0060)**	-0.1378 (± 0.0062)	-0.1642 (± 0.0059)	-0.1390 (± 0.0069)	-0.1453 (± 0.0053)	-0.1605 (± 0.0055)	-0.1581 (± 0.0065)	-0.1517 (± 0.0057)	-0.1449 (± 0.0049)	-0.1357 (± 0.0045)	-0.1359 (± 0.0049)
us_crime	359945 (42730)	-0.1301 (± 0.0061)	-0.1304 (± 0.0054)	-0.1459 (± 0.0114)	-0.1305 (± 0.0053)	-0.1346 (± 0.0074)	-0.1360 (± 0.0083)	-0.1621 (± 0.0537)	-0.1339 (± 0.0071)	-0.1302 (± 0.0049)	**-0.1253 (± 0.0052)**	-0.1265 (± 0.0049)
MIP2016regression	360945 (43071)	-20560.6384 (± 1646.3594)	-21449.9103 (± 1612.2797)	-25957.1756 (± 2570.8231)	-20583.7643 (± 1439.9461)	-22929.1425 (± 2192.7170)	-25180.3342 (± 1686.8821)	-24861.1096 (± 2376.9443)	-31791.5722 (± 2143.6984)	-20838.3065 (± 1724.7616)	**-19738.0161 (± 2032.4651)**	-20277.5602 (± 2195.3958)
airfoil_self_noise	361235 (44957)	-1.0280 (± 0.1326)	-1.0953 (± 0.1700)	-2.1344 (± 0.2280)	-1.3636 (± 0.1996)	-1.6729 (± 0.1884)	-4.8235 (± 0.2662)	-4.4687 (± 0.8659)	-3.2005 (± 0.3349)	-1.2050 (± 0.2029)	-0.8748 (± 0.1355)	**-0.8598 (± 0.1254)**
auction_verification	361236 (44958)	**-241.2112 (± 56.4365)**	-511.0889 (± 64.1043)	-3383.0746 (± 529.2227)	-398.1578 (± 62.2005)	-751.3872 (± 117.8477)	-6185.0765 (± 356.1370)	-2902.3171 (± 773.6211)	-5465.7424 (± 293.9870)	-341.1392 (± 72.5863)	-377.8745 (± 47.3536)	-397.0643 (± 62.2035)
concrete_compressive_strength	361237 (44959)	-3.6902 (± 0.7659)	-3.6890 (± 0.6452)	-9.0353 (± 0.9600)	-3.9162 (± 0.6132)	-4.8460 (± 0.6867)	-10.4353 (± 0.5271)	-7.9273 (± 1.0742)	-6.2532 (± 0.5861)	-3.8371 (± 0.7216)	**-3.6562 (± 0.8653)**	-3.6655 (± 0.9147)
energy_efficiency	361617 (44960)	**-0.2606 (± 0.0663)**	-0.2617 (± 0.0730)	-2.6296 (± 0.1971)	-0.3143 (± 0.0634)	-0.4585 (± 0.0816)	-2.9429 (± 0.2484)	-2.9727 (± 0.2978)	-1.0502 (± 0.1681)	-0.2784 (± 0.0659)	-0.3722 (± 0.0668)	-0.3563 (± 0.0642)
geographical_origin_of_music	361243 (44965)	-14.8767 (± 1.9262)	-15.2125 (± 1.7538)	-16.0680 (± 1.5899)	-15.5394 (± 1.5841)	-15.8007 (± 1.6131)	-16.6821 (± 1.2865)	-17.6265 (± 1.5550)	-15.7064 (± 1.8521)	-15.2186 (± 1.5773)	**-14.3053 (± 2.0271)**	-14.4734 (± 2.0161)
student_performance_por	361619 (44967)	-2.6533 (± 0.5596)	-2.6968 (± 0.5447)	-2.9864 (± 0.5625)	-2.6883 (± 0.4981)	-2.6743 (± 0.5265)	-2.7538 (± 0.4983)	-2.7606 (± 0.4923)	-2.7300 (± 0.5745)	-2.6564 (± 0.5078)	**-2.6362 (± 0.5266)**	-2.6372 (± 0.5281)
QSAR_fish_toxicity	361621 (44970)	-0.8706 (± 0.0600)	-0.8736 (± 0.0638)	-0.8898 (± 0.0592)	-0.9020 (± 0.0717)	-0.8637 (± 0.0647)	-0.9533 (± 0.0890)	-0.9442 (± 0.0840)	-0.8891 (± 0.0594)	-0.8692 (± 0.0598)	**-0.8476 (± 0.0622)**	-0.8560 (± 0.0680)
grid_stability	361251 (44973)	**-0.0041 (± 0.0003)**	-0.0064 (± 0.0002)	-0.0165 (± 0.0005)	-0.0072 (± 0.0001)	-0.0117 (± 0.0003)	-0.0220 (± 0.0007)	-0.0169 (± 0.0019)	-0.0369 (± 0.0008)	-0.0072 (± 0.0004)	-0.0047 (± 0.0003)	-0.0047 (± 0.0003)
cpu_activity	361256 (44978)	-2.0489 (± 0.0877)	-2.1149 (± 0.1343)	-2.8326 (± 0.1109)	-2.2425 (± 0.2845)	-2.4535 (± 0.1801)	-9.6325 (± 0.9048)	-4.4565 (± 4.9866)	-2.5600 (± 0.1394)	-2.1289 (± 0.1017)	**-2.0097 (± 0.0956)**	-2.0452 (± 0.1366)
kin8nm	361258 (44980)	**-0.0626 (± 0.0015)**	-0.0901 (± 0.0030)	-0.1152 (± 0.0025)	-0.1060 (± 0.0024)	-0.1391 (± 0.0037)	-0.2019 (± 0.0036)	-0.1066 (± 0.0057)	-0.0772 (± 0.0020)	-0.1050 (± 0.0031)	-0.0660 (± 0.0025)	-0.0661 (± 0.0024)
pumadyn32nh	361259 (44981)	-0.0209 (± 0.0007)	-0.0212 (± 0.0007)	-0.0338 (± 0.0010)	-0.0217 (± 0.0007)	-0.0216 (± 0.0008)	-0.0330 (± 0.0008)	-0.0344 (± 0.0013)	-0.0348 (± 0.0008)	-0.0215 (± 0.0007)	**-0.0206 (± 0.0007)**	-0.0207 (± 0.0007)
cars	361622 (44994)	-2080.5064 (± 181.5751)	-2069.9186 (± 211.7686)	-2534.9766 (± 246.4226)	-2144.4847 (± 221.2022)	-2211.9957 (± 177.6526)	-2945.7366 (± 317.5045)	-2168.9747 (± 155.5099)	-5496.2717 (± 1573.3142)	-2115.3212 (± 238.9856)	**-1981.8505 (± 180.1296)**	-2023.9633 (± 224.0425)
tecator	359934 (505)	-0.6825 (± 0.1370)	-1.3546 (± 0.4763)	-4.7739 (± 1.2720)	-1.7078 (± 0.5893)	-1.3940 (± 0.2233)	-0.8571 (± 0.3316)	-11.0025 (± 7.4151)	-1.3195 (± 0.6353)	-0.9707 (± 0.2468)	-0.4612 (± 0.1415)	**-0.4453 (± 0.1122)**
space_ga	359933 (507)	-0.0932 (± 0.0165)	-0.1041 (± 0.0164)	-0.1216 (± 0.0208)	-0.1030 (± 0.0130)	-0.1154 (± 0.0215)	-0.1286 (± 0.0089)	-0.1438 (± 0.0208)	-0.1032 (± 0.0106)	-0.1066 (± 0.0168)	-0.0915 (± 0.0144)	**-0.0913 (± 0.0155)**
boston	359950 (531)	-2.7009 (± 0.7868)	-2.8746 (± 0.9943)	-4.5966 (± 1.2450)	-3.1347 (± 0.9418)	-3.2505 (± 1.0082)	-4.7527 (± 1.1546)	-4.9578 (± 2.0271)	-3.4990 (± 1.2043)	-2.9128 (± 0.7930)	**-2.5158 (± 0.7181)**	-2.5800 (± 0.7348)
socmob	359932 (541)	-11.8806 (± 6.7059)	-15.1392 (± 8.1160)	-17.4632 (± 7.0051)	-14.5292 (± 3.8946)	-18.5558 (± 6.3245)	-24.8820 (± 12.4281)	-20.4125 (± 15.2854)	-21.6415 (± 8.1913)	-12.5944 (± 4.4016)	-11.9149 (± 6.8727)	**-10.0660 (± 4.4118)**
sensory	359931 (546)	-0.6833 (± 0.0610)	-0.7324 (± 0.0487)	-0.7476 (± 0.0722)	-0.6854 (± 0.0623)	-0.6955 (± 0.0635)	-0.7644 (± 0.0745)	-0.7576 (± 0.0726)	-0.7103 (± 0.0570)	-0.6871 (± 0.0529)	**-0.6832 (± 0.0637)**	-0.6839 (± 0.0582)
quake	359930 (550)	**-0.1882 (± 0.0097)**	-0.1884 (± 0.0102)	-0.1930 (± 0.0097)	-0.1895 (± 0.0104)	-0.1885 (± 0.0094)	-0.1888 (± 0.0103)	-0.1900 (± 0.0110)	-0.1959 (± 0.0102)	-0.1885 (± 0.0094)	-0.1889 (± 0.0102)	-0.1902 (± 0.0112)

Supplementary Table 3: **Raw Performance Scores per Dataset for the Grinsztajn Medium-Sized Benchmark.** The table presents the mean and standard deviation of the ROC AUC score across all folds per dataset for each compared method after four hours. The second column details the OpenML tasks and dataset IDs.

Dataset Name	Task (Dataset)	AutoGluon	CatBoost	KNN	LightGBM	RF	Logistic Regression	MLP	SVM	XGB	TabPFN (PHE)	TabPFN
credit	361055 (44089)	0.8578 (± 0.0069)	0.8578 (± 0.0073)	0.6975 (± 0.0105)	0.8561 (± 0.0079)	0.8545 (± 0.0073)	0.7987 (± 0.0121)	0.8239 (± 0.0111)	0.8131 (± 0.0116)	0.8573 (± 0.0078)	**0.8582 (± 0.0071)**	0.8575 (± 0.0070)
california	361056 (44090)	0.9712 (± 0.0036)	0.9673 (± 0.0041)	0.9172 (± 0.0063)	0.9665 (± 0.0039)	0.9576 (± 0.0052)	0.9068 (± 0.0082)	0.9208 (± 0.0091)	0.9319 (± 0.0087)	0.9671 (± 0.0041)	**0.9757 (± 0.0032)**	0.9745 (± 0.0033)
wine	361057 (44091)	**0.9191 (± 0.0145)**	0.9077 (± 0.0227)	0.8326 (± 0.0160)	0.9052 (± 0.0167)	0.9072 (± 0.0225)	0.8090 (± 0.0259)	0.8391 (± 0.0259)	0.8556 (± 0.0208)	0.9171 (± 0.0167)	0.9107 (± 0.0204)	0.9083 (± 0.0190)
electricity	361060 (44120)	0.9492 (± 0.0037)	0.9402 (± 0.0049)	0.8283 (± 0.0068)	0.9394 (± 0.0039)	0.9279 (± 0.0038)	0.8187 (± 0.0062)	0.8496 (± 0.0071)	0.8649 (± 0.0066)	0.9419 (± 0.0038)	**0.9572 (± 0.0027)**	0.9541 (± 0.0032)
covertype	361061 (44121)	0.9228 (± 0.0018)	0.8998 (± 0.0009)	0.8520 (± 0.0026)	0.9008 (± 0.0015)	0.8924 (± 0.0017)	0.6629 (± 0.0036)	0.8407 (± 0.0007)	0.8497 (± 0.0018)	0.9045 (± 0.0011)	**0.9390 (± 0.0016)**	0.9356 (± 0.0027)
pol	361062 (44122)	**0.9997 (± 0.0002)**	0.9995 (± 0.0003)	0.9932 (± 0.0019)	0.9993 (± 0.0003)	0.9989 (± 0.0005)	0.9416 (± 0.0050)	0.9992 (± 0.0004)	0.9990 (± 0.0006)	0.9994 (± 0.0003)	0.9997 (± 0.0001)	0.9997 (± 0.0001)
house_16H	361063 (44123)	0.9575 (± 0.0028)	0.9559 (± 0.0037)	0.9063 (± 0.0055)	0.9538 (± 0.0038)	0.9486 (± 0.0043)	0.8912 (± 0.0082)	0.9383 (± 0.0048)	0.9346 (± 0.0040)	0.9554 (± 0.0039)	**0.9590 (± 0.0033)**	0.9582 (± 0.0030)
kdd_ipums_la_97small	361064 (44124)	0.9488 (± 0.0099)	0.9493 (± 0.0096)	0.9031 (± 0.0086)	0.9491 (± 0.0102)	0.9487 (± 0.0093)	0.9278 (± 0.0126)	0.9398 (± 0.0095)	0.9262 (± 0.0126)	0.9492 (± 0.0110)	**0.9496 (± 0.0103)**	0.9489 (± 0.0107)
MagicTelescope	361065 (44125)	0.9457 (± 0.0063)	0.9378 (± 0.0076)	0.8968 (± 0.0076)	0.9351 (± 0.0067)	0.9335 (± 0.0058)	0.8426 (± 0.0088)	0.9179 (± 0.0095)	0.9213 (± 0.0070)	0.9374 (± 0.0073)	**0.9479 (± 0.0055)**	0.9477 (± 0.0054)
bankmarketing	361066 (44126)	0.8894 (± 0.0064)	0.8874 (± 0.0067)	0.8583 (± 0.0102)	0.8877 (± 0.0060)	0.8825 (± 0.0065)	0.8316 (± 0.0083)	0.8697 (± 0.0075)	0.8690 (± 0.0061)	0.8877 (± 0.0060)	**0.8915 (± 0.0068)**	0.8896 (± 0.0068)
phoneme	361067 (44127)	0.9616 (± 0.0076)	0.9557 (± 0.0092)	0.9212 (± 0.0147)	0.9527 (± 0.0060)	0.9565 (± 0.0092)	0.8111 (± 0.0338)	0.8747 (± 0.0246)	0.9200 (± 0.0124)	0.9590 (± 0.0082)	**0.9633 (± 0.0085)**	0.9627 (± 0.0087)
MiniBooNE	361068 (44128)	**0.9847 (± 0.0014)**	0.9826 (± 0.0014)	0.9319 (± 0.0035)	0.9819 (± 0.0015)	0.9748 (± 0.0016)	0.9074 (± 0.0045)	0.8898 (± 0.0039)	0.9647 (± 0.0021)	0.9828 (± 0.0014)	0.9844 (± 0.0014)	0.9837 (± 0.0013)
Higgs	361069 (44129)	0.7982 (± 0.0013)	0.7910 (± 0.0013)	0.5605 (± 0.0036)	0.7893 (± 0.0014)	0.7780 (± 0.0014)	0.6791 (± 0.0015)	0.7281 (± 0.0031)	0.6925 (± 0.0025)	0.7925 (± 0.0015)	**0.8014 (± 0.0014)**	0.7996 (± 0.0020)
eye_movements	361070 (44130)	0.8165 (± 0.0143)	0.7556 (± 0.0176)	0.5935 (± 0.0156)	0.7623 (± 0.0175)	0.7066 (± 0.0134)	0.5857 (± 0.0152)	0.6382 (± 0.0174)	0.6336 (± 0.0152)	0.7770 (± 0.0175)	**0.8958 (± 0.0135)**	0.8616 (± 0.0151)
jannis	361071 (44131)	0.8651 (± 0.0032)	0.8579 (± 0.0042)	0.7583 (± 0.0065)	0.8547 (± 0.0038)	0.8461 (± 0.0035)	0.8038 (± 0.0040)	0.8261 (± 0.0032)	0.8170 (± 0.0023)	0.8600 (± 0.0035)	**0.8712 (± 0.0025)**	0.8704 (± 0.0030)
electricity	361110 (44156)	0.9638 (± 0.0033)	0.9496 (± 0.0039)	0.8138 (± 0.0075)	0.9559 (± 0.0036)	0.9409 (± 0.0037)	0.8270 (± 0.0060)	0.8599 (± 0.0060)	0.8695 (± 0.0052)	0.9611 (± 0.0025)	0.9735 (± 0.0021)	**0.9742 (± 0.0022)**
eye_movements	361111 (44157)	0.8288 (± 0.0128)	0.7633 (± 0.0179)	0.6192 (± 0.0205)	0.7732 (± 0.0080)	0.7174 (± 0.0115)	0.5999 (± 0.0181)	0.6442 (± 0.0136)	0.6505 (± 0.0119)	0.7817 (± 0.0164)	**0.8888 (± 0.0163)**	0.8307 (± 0.0267)
KDDCup09_upselling	361112 (44158)	0.9059 (± 0.0133)	0.9064 (± 0.0142)	0.7703 (± 0.0134)	0.9069 (± 0.0148)	0.8994 (± 0.0166)	0.8396 (± 0.0170)	0.8381 (± 0.0112)	0.8372 (± 0.0163)	0.9044 (± 0.0154)	**0.9088 (± 0.0129)**	0.9077 (± 0.0125)
covertype	361113 (44159)	0.9586 (± 0.0009)	0.9388 (± 0.0014)	0.8970 (± 0.0032)	0.9360 (± 0.0019)	0.9267 (± 0.0010)	0.8475 (± 0.0015)	0.8679 (± 0.0026)	0.8696 (± 0.0013)	0.9397 (± 0.0016)	**0.9644 (± 0.0015)**	0.9615 (± 0.0019)
rl	361114 (44160)	0.9214 (± 0.0143)	0.8965 (± 0.0166)	0.7339 (± 0.0291)	0.8771 (± 0.0173)	0.8582 (± 0.0235)	0.6695 (± 0.0299)	0.7387 (± 0.0208)	0.7232 (± 0.0151)	0.8637 (± 0.0139)	**0.9460 (± 0.0142)**	0.9405 (± 0.0155)
roadsafety	361115 (44161)	**0.8844 (± 0.0032)**	0.8580 (± 0.0036)	0.7456 (± 0.0041)	0.8532 (± 0.0045)	0.8453 (± 0.0036)	0.7510 (± 0.0049)	0.8317 (± 0.0050)	0.7868 (± 0.0050)	0.8564 (± 0.0040)	0.8718 (± 0.0020)	0.8697 (± 0.0020)
compass	361116 (44162)	**0.8924 (± 0.0075)**	0.8364 (± 0.0077)	0.7347 (± 0.0123)	0.8527 (± 0.0079)	0.8394 (± 0.0079)	0.7486 (± 0.0126)	0.7851 (± 0.0126)	0.7804 (± 0.0145)	0.8443 (± 0.0165)	0.8758 (± 0.0124)	0.8708 (± 0.0067)

Supplementary Table 4: **Raw Performance Scores per Dataset for the TabZilla Benchmark.** The table presents the mean and standard deviation of the ROC AUC score across all folds per dataset for each compared method after one hour. The second column details the OpenML tasks and dataset IDs. The second column details the OpenMLtask and dataset IDs.

Dataset Name	Task (Dataset)	AutoGluon	CatBoost	KNN	LightGBM	RF	Linear Regression	MLP	SVM	XGB	TabPFN (PHE)	TabPFN
ada_agnostic	3896 (1043)	0.9105 (± 0.0152)	0.9070 (± 0.0145)	0.8537 (± 0.0178)	0.9077 (± 0.0138)	0.9013 (± 0.0141)	0.8969 (± 0.0145)	0.8863 (± 0.0175)	0.8838 (± 0.0129)	**0.9107 (± 0.0136)**	0.9059 (± 0.0139)	0.9060 (± 0.0138)
pc4	3902 (1049)	0.9527 (± 0.0195)	0.9548 (± 0.0176)	0.8439 (± 0.0505)	0.9452 (± 0.0220)	0.9416 (± 0.0201)	0.9065 (± 0.0306)	0.9198 (± 0.0319)	0.9053 (± 0.0362)	0.9495 (± 0.0164)	**0.9589 (± 0.0164)**	0.9550 (± 0.0202)
pc3	3903 (1050)	**0.8659 (± 0.0382)**	0.8501 (± 0.0348)	0.7911 (± 0.0604)	0.8432 (± 0.0340)	0.8490 (± 0.0396)	0.7907 (± 0.0419)	0.8220 (± 0.0390)	0.7299 (± 0.0583)	0.8604 (± 0.0380)	0.8584 (± 0.0371)	0.8618 (± 0.0372)
kc2	3913 (1063)	0.8360 (± 0.0931)	0.8431 (± 0.0972)	0.8154 (± 0.0888)	0.8423 (± 0.0965)	0.8429 (± 0.0828)	0.8406 (± 0.0880)	0.6788 (± 0.2587)	0.8446 (± 0.0881)	**0.8460 (± 0.0914)**	0.8323 (± 0.0933)	0.8358 (± 0.0924)
kc1	3917 (1067)	0.8422 (± 0.0299)	0.8265 (± 0.0392)	0.7619 (± 0.0509)	0.8060 (± 0.0361)	0.8317 (± 0.0368)	0.7989 (± 0.0358)	0.8025 (± 0.0354)	0.7985 (± 0.0347)	0.8306 (± 0.0365)	**0.8502 (± 0.0284)**	0.8481 (± 0.0317)
pc1	3918 (1068)	0.8953 (± 0.0417)	0.8791 (± 0.0603)	0.8064 (± 0.0774)	0.8663 (± 0.0561)	0.8579 (± 0.0475)	0.8299 (± 0.0569)	0.8076 (± 0.0540)	0.7369 (± 0.1141)	0.8803 (± 0.0537)	**0.9092 (± 0.0412)**	0.9066 (± 0.0390)
musk	3950 (1116)	1.0000 (± 0.0001)	**1.0000 (± 0.0000)**	0.9956 (± 0.0019)	**1.0000 (± 0.0000)**	**1.0000 (± 0.0000)**	**1.0000 (± 0.0000)**	**1.0000 (± 0.0000)**	**1.0000 (± 0.0000)**	0.7418 (± 0.0746)	0.9997 (± 0.0011)	**1.0000 (± 0.0000)**
balancescale	11 (11)	0.9983 (± 0.0035)	0.9586 (± 0.0176)	0.9078 (± 0.0350)	0.9989 (± 0.0019)	0.8549 (± 0.0337)	0.9726 (± 0.0130)	0.9809 (± 0.0090)	0.9983 (± 0.0030)	0.9985 (± 0.0026)	**1.0000 (± 0.0000)**	**1.0000 (± 0.0000)**
mfeatfactors	12 (12)	0.9993 (± 0.0009)	0.9988 (± 0.0016)	0.9969 (± 0.0027)	0.9990 (± 0.0010)	0.9984 (± 0.0020)	0.9985 (± 0.0016)	0.9988 (± 0.0016)	0.9995 (± 0.0007)	0.9990 (± 0.0013)	0.9997 (± 0.0004)	**0.9997 (± 0.0004)**
breastcancer	145799 (13)	0.7119 (± 0.0961)	0.6963 (± 0.0957)	0.6447 (± 0.0830)	0.6734 (± 0.1218)	0.7233 (± 0.1080)	0.6830 (± 0.0995)	0.6230 (± 0.1506)	0.7254 (± 0.0989)	0.7121 (± 0.1132)	0.7216 (± 0.0887)	**0.7381 (± 0.0970)**
acuteinflammations	10089 (1455)	**1.0000 (± 0.0000)**	**1.0000 (± 0.0000)**	**1.0000 (± 0.0000)**	**1.0000 (± 0.0000)**	**1.0000 (± 0.0000)**	**1.0000 (± 0.0000)**	**1.0000 (± 0.0000)**	**1.0000 (± 0.0000)**	**1.0000 (± 0.0000)**	**1.0000 (± 0.0000)**	**1.0000 (± 0.0000)**
banknoteauthentication	10093 (1462)	**1.0000 (± 0.0000)**	**1.0000 (± 0.0000)**	0.9987 (± 0.0028)	**1.0000 (± 0.0000)**	0.9999 (± 0.0002)	0.9998 (± 0.0004)	0.9998 (± 0.0005)	**1.0000 (± 0.0000)**	**1.0000 (± 0.0000)**	**1.0000 (± 0.0000)**	**1.0000 (± 0.0000)**
bloodtransfusionservicecenter	10101 (1464)	0.7448 (± 0.0580)	0.7570 (± 0.0543)	0.7292 (± 0.0595)	0.7381 (± 0.0509)	0.7368 (± 0.0556)	0.7593 (± 0.0434)	0.6585 (± 0.1373)	0.7364 (± 0.0501)	0.7556 (± 0.0446)	**0.7621 (± 0.0447)**	0.7615 (± 0.0409)
cardiotocography	9979 (1466)	**1.0000 (± 0.0000)**	**1.0000 (± 0.0000)**	**1.0000 (± 0.0000)**	**1.0000 (± 0.0000)**	**1.0000 (± 0.0000)**	**1.0000 (± 0.0000)**	**1.0000 (± 0.0000)**	**1.0000 (± 0.0000)**	**1.0000 (± 0.0000)**	**1.0000 (± 0.0000)**	**1.0000 (± 0.0000)**
fertility	9984 (1473)	0.5667 (± 0.3646)	0.5188 (± 0.3248)	0.6733 (± 0.2230)	0.6858 (± 0.2759)	0.5826 (± 0.2830)	0.4042 (± 0.2946)	0.5674 (± 0.2456)	0.5333 (± 0.2981)	**0.6934 (± 0.3310)**	0.5458 (± 0.3544)	0.5576 (± 0.2688)
firstordertheoremproving	9985 (1475)	**0.8611 (± 0.0097)**	0.8510 (± 0.0089)	0.7942 (± 0.0115)	0.8516 (± 0.0101)	0.8523 (± 0.0096)	0.7097 (± 0.0129)	0.7788 (± 0.0107)	0.7550 (± 0.0310)	0.8554 (± 0.0079)	0.8609 (± 0.0081)	0.8580 (± 0.0085)
hillvalley	145847 (1479)	0.9754 (± 0.0135)	0.6307 (± 0.0372)	0.5920 (± 0.0298)	0.6583 (± 0.0371)	0.6036 (± 0.0416)	0.9284 (± 0.0425)	0.6702 (± 0.0747)	0.9027 (± 0.0553)	0.7054 (± 0.0425)	**1.0000 (± 0.0000)**	0.9999 (± 0.0002)
ilpd	9971 (1480)	0.7481 (± 0.0537)	0.7421 (± 0.0692)	0.6740 (± 0.0548)	0.7154 (± 0.0629)	0.7237 (± 0.0428)	0.7552 (± 0.0677)	0.7557 (± 0.0631)	0.6799 (± 0.0547)	0.7193 (± 0.0706)	**0.7595 (± 0.0544)**	0.7586 (± 0.0634)
ozonelevel8hr	9978 (1487)	0.9336 (± 0.0192)	0.9313 (± 0.0190)	0.8691 (± 0.0508)	0.9233 (± 0.0252)	0.9118 (± 0.0345)	0.9148 (± 0.0295)	0.9217 (± 0.0254)	0.9212 (± 0.0264)	0.9314 (± 0.0179)	**0.9352 (± 0.0184)**	0.9272 (± 0.0217)
phoneme	9952 (1489)	0.9733 (± 0.0076)	0.9634 (± 0.0092)	0.9319 (± 0.0137)	0.9597 (± 0.0094)	0.9635 (± 0.0097)	0.8136 (± 0.0218)	0.8916 (± 0.0117)	0.9103 (± 0.0117)	0.9640 (± 0.0087)	**0.9733 (± 0.0073)**	0.9724 (± 0.0075)
qsarbiodeg	9957 (1494)	**0.9421 (± 0.0316)**	0.9287 (± 0.0395)	0.9056 (± 0.0344)	0.9276 (± 0.0425)	0.9333 (± 0.0309)	0.9247 (± 0.0321)	0.9272 (± 0.0323)	0.9225 (± 0.0357)	0.9345 (± 0.0384)	0.9409 (± 0.0311)	0.9386 (± 0.0322)
wallrobotnavigation	9960 (1497)	**1.0000 (± 0.0000)**	1.0000 (± 0.0000)	0.9653 (± 0.0050)	0.9999 (± 0.0002)	0.9999 (± 0.0000)	0.9085 (± 0.0068)	0.9908 (± 0.0015)	0.9904 (± 0.0019)	1.0000 (± 0.0000)	1.0000 (± 0.0000)	1.0000 (± 0.0000)
mfeatfourier	14 (14)	0.9878 (± 0.0037)	0.9852 (± 0.0037)	0.9757 (± 0.0068)	0.9842 (± 0.0038)	0.9830 (± 0.0032)	0.9814 (± 0.0043)	0.9795 (± 0.0053)	0.9860 (± 0.0039)	0.9834 (± 0.0042)	**0.9921 (± 0.0026)**	0.9908 (± 0.0022)
semeion	9964 (1501)	0.9986 (± 0.0009)	0.9985 (± 0.0009)	0.9938 (± 0.0029)	0.9974 (± 0.0017)	0.9966 (± 0.0013)	0.9957 (± 0.0026)	0.9962 (± 0.0019)	**0.9990 (± 0.0006)**	0.9980 (± 0.0016)	0.9987 (± 0.0006)	0.9984 (± 0.0009)
wdbc	9946 (1510)	**0.9958 (± 0.0070)**	0.9947 (± 0.0081)	0.9883 (± 0.0137)	0.9939 (± 0.0081)	0.9896 (± 0.0135)	0.9942 (± 0.0088)	0.9898 (± 0.0155)	0.9887 (± 0.0165)	0.9945 (± 0.0069)	0.9958 (± 0.0080)	0.9950 (± 0.0087)
bankmarketing	9899 (1558)	0.9188 (± 0.0149)	0.9167 (± 0.0171)	0.7474 (± 0.0333)	0.9094 (± 0.0163)	0.9073 (± 0.0162)	0.8904 (± 0.0259)	0.8765 (± 0.0187)	0.8855 (± 0.0216)	0.8927 (± 0.0511)	0.9293 (± 0.0132)	**0.9298 (± 0.0115)**
breastw	15 (15)	0.9948 (± 0.0048)	**0.9961 (± 0.0047)**	0.9903 (± 0.0087)	0.9903 (± 0.0083)	0.9922 (± 0.0074)	0.9948 (± 0.0053)	0.9946 (± 0.0065)	0.9955 (± 0.0046)	0.9931 (± 0.0065)	0.9952 (± 0.0041)	0.9950 (± 0.0051)
mfeatkarhunen	16 (16)	0.9996 (± 0.0002)	0.9994 (± 0.0004)	0.9965 (± 0.0023)	0.9988 (± 0.0009)	0.9982 (± 0.0010)	0.9972 (± 0.0016)	0.9973 (± 0.0015)	0.9996 (± 0.0003)	0.9991 (± 0.0005)	**0.9997 (± 0.0004)**	0.9997 (± 0.0003)
satimage	2074 (182)	0.9944 (± 0.0013)	0.9923 (± 0.0020)	0.9861 (± 0.0029)	0.9916 (± 0.0020)	0.9899 (± 0.0028)	0.9765 (± 0.0056)	0.9850 (± 0.0031)	0.9903 (± 0.0021)	0.9924 (± 0.0020)	**0.9955 (± 0.0013)**	0.9951 (± 0.0015)
eucalyptus	2079 (188)	0.9349 (± 0.0170)	0.9160 (± 0.0192)	0.8439 (± 0.0153)	0.9131 (± 0.0183)	0.9146 (± 0.0200)	0.9138 (± 0.0180)	0.9082 (± 0.0150)	0.9061 (± 0.0134)	0.9075 (± 0.0218)	**0.9365 (± 0.0151)**	0.9348 (± 0.0144)
mfeatmorphological	18 (18)	**0.9774 (± 0.0065)**	0.9652 (± 0.0072)	0.9562 (± 0.0113)	0.9637 (± 0.0076)	0.9629 (± 0.0082)	0.9660 (± 0.0076)	0.9623 (± 0.0073)	0.9681 (± 0.0081)	0.9629 (± 0.0068)	0.9698 (± 0.0061)	0.9686 (± 0.0075)
anneal	2867 (1)	0.9196 (± 0.0423)	0.9999 (± 0.0005)	0.9838 (± 0.0329)	0.9980 (± 0.0053)	0.9997 (± 0.0008)	0.9810 (± 0.0355)	0.9938 (± 0.0100)	0.9935 (± 0.0176)	0.9972 (± 0.0080)	0.9996 (± 0.0009)	**1.0000 (± 0.0001)**
mfeatzernike	22 (22)	**0.9953 (± 0.0014)**	0.9768 (± 0.0027)	0.9781 (± 0.0041)	0.9743 (± 0.0034)	0.9751 (± 0.0038)	0.9815 (± 0.0018)	0.9819 (± 0.0022)	0.9920 (± 0.0016)	0.9755 (± 0.0035)	0.9900 (± 0.0023)	0.9854 (± 0.0017)
cjs	14967 (23380)	**1.0000 (± 0.0000)**	**1.0000 (± 0.0000)**	0.8777 (± 0.0114)	**1.0000 (± 0.0000)**	0.9600 (± 0.0073)	0.7120 (± 0.0174)	0.8011 (± 0.0102)	0.8028 (± 0.0118)	0.8447 (± 0.0878)	**1.0000 (± 0.0000)**	**1.0000 (± 0.0000)**
dressessales	125920 (23381)	0.6054 (± 0.0565)	0.6125 (± 0.0619)	0.5474 (± 0.0740)	0.6155 (± 0.0658)	0.5906 (± 0.0827)	0.6296 (± 0.0610)	0.5209 (± 0.1281)	**0.6447 (± 0.0977)**	0.5626 (± 0.0865)	0.5934 (± 0.0770)	0.6245 (± 0.0784)
cmc	23 (23)	0.7472 (± 0.0305)	0.7464 (± 0.0305)	0.6377 (± 0.0313)	0.7461 (± 0.0390)	0.7463 (± 0.0329)	0.7028 (± 0.0407)	0.6979 (± 0.0336)	0.6850 (± 0.0399)	0.7429 (± 0.0351)	**0.7557 (± 0.0322)**	0.7532 (± 0.0287)
mushroom	24 (24)	**1.0000 (± 0.0000)**	**1.0000 (± 0.0000)**	**1.0000 (± 0.0000)**	**1.0000 (± 0.0000)**	**1.0000 (± 0.0000)**	**1.0000 (± 0.0000)**	**1.0000 (± 0.0000)**	**1.0000 (± 0.0000)**	**1.0000 (± 0.0000)**	**1.0000 (± 0.0000)**	**1.0000 (± 0.0000)**
colic	25 (25)	0.9262 (± 0.0374)	0.9306 (± 0.0349)	0.8548 (± 0.0827)	0.9178 (± 0.0447)	0.9139 (± 0.0461)	0.8807 (± 0.0747)	0.8608 (± 0.0870)	0.8782 (± 0.0668)	0.6636 (± 0.1232)	**0.9412 (± 0.0350)**	0.9375 (± 0.0310)
colic	27 (27)	0.8913 (± 0.0580)	0.8986 (± 0.0708)	0.8403 (± 0.0921)	0.8876 (± 0.0519)	0.8962 (± 0.0635)	0.8769 (± 0.0607)	0.8625 (± 0.0636)	0.8664 (± 0.0843)	0.8899 (± 0.0627)	0.8875 (± 0.0597)	**0.9047 (± 0.0510)**
optdigits	28 (28)	0.9999 (± 0.0001)	0.9999 (± 0.0001)	0.9992 (± 0.0010)	0.9998 (± 0.0002)	0.9996 (± 0.0002)	0.9993 (± 0.0004)	0.9997 (± 0.0002)	**0.9999 (± 0.0001)**	0.9999 (± 0.0001)	0.9999 (± 0.0001)	0.9999 (± 0.0001)
creditapproval	29 (29)	0.9380 (± 0.0341)	0.9383 (± 0.0312)	0.9096 (± 0.0402)	0.9398 (± 0.0355)	0.9321 (± 0.0464)	0.9281 (± 0.0365)	0.9167 (± 0.0472)	0.9188 (± 0.0407)	0.9352 (± 0.0374)	**0.9413 (± 0.0391)**	0.9410 (± 0.0355)
pageblocks	30 (30)	**0.9949 (± 0.0027)**	0.9940 (± 0.0032)	0.9639 (± 0.0171)	0.9932 (± 0.0032)	0.9913 (± 0.0077)	0.9823 (± 0.0080)	0.9863 (± 0.0078)	0.9873 (± 0.0054)	0.9944 (± 0.0026)	0.9943 (± 0.0035)	0.9947 (± 0.0035)
scene	3485 (312)	**0.9931 (± 0.0071)**	0.9884 (± 0.0127)	0.9834 (± 0.0139)	0.9886 (± 0.0134)	0.9472 (± 0.0189)	0.9925 (± 0.0081)	0.9857 (± 0.0185)	0.9890 (± 0.0116)	0.9883 (± 0.0123)	0.9916 (± 0.0094)	0.9922 (± 0.0089)
creditg	31 (31)	0.7956 (± 0.0350)	0.7970 (± 0.0396)	0.7431 (± 0.0298)	0.7782 (± 0.0447)	0.7935 (± 0.0344)	0.7870 (± 0.0412)	0.6759 (± 0.0800)	0.7883 (± 0.0232)	0.7949 (± 0.0365)	0.7945 (± 0.0331)	**0.8006 (± 0.0419)**
hayesroth	146063 (329)	0.9620 (± 0.0354)	0.9631 (± 0.0339)	0.8758 (± 0.0613)	0.9664 (± 0.0319)	0.9506 (± 0.0381)	0.7837 (± 0.1028)	0.7694 (± 0.0771)	0.9359 (± 0.0410)	**0.9729 (± 0.0394)**	**0.9729 (± 0.0302)**	0.9674 (± 0.0398)
monksproblems2	146065 (334)	**1.0000 (± 0.0000)**	0.9539 (± 0.0309)	0.7967 (± 0.0474)	**1.0000 (± 0.0000)**	0.9946 (± 0.0059)	0.5252 (± 0.0937)	**1.0000 (± 0.0000)**	**1.0000 (± 0.0000)**	**1.0000 (± 0.0000)**	**1.0000 (± 0.0000)**	**1.0000 (± 0.0000)**
dermatology	35 (35)	0.9989 (± 0.0019)	0.9985 (± 0.0023)	0.9990 (± 0.0015)	0.9978 (± 0.0029)	0.9989 (± 0.0019)	0.9987 (± 0.0022)	0.9986 (± 0.0021)	0.9986 (± 0.0024)	0.9990 (± 0.0021)	**0.9990 (± 0.0019)**	0.9989 (± 0.0021)
JapaneseVowels	3510 (375)	1.0000 (± 0.0001)	0.9999 (± 0.0001)	0.9994 (± 0.0004)	0.9998 (± 0.0001)	0.9994 (± 0.0002)	0.9972 (± 0.0006)	0.9985 (± 0.0005)	0.9999 (± 0.0001)	0.9999 (± 0.0001)	**1.0000 (± 0.0000)**	1.0000 (± 0.0000)
synthetic_control	3512 (377)	**1.0000 (± 0.0000)**	0.9997 (± 0.0006)	0.9995 (± 0.0008)	0.9994 (± 0.0010)	0.9997 (± 0.0006)	0.9997 (± 0.0003)	0.9990 (± 0.0007)	0.9999 (± 0.0002)	0.9998 (± 0.0005)	**1.0000 (± 0.0000)**	**1.0000 (± 0.0000)**
diabetes	37 (37)	0.8365 (± 0.0518)	0.8427 (± 0.0439)	0.8054 (± 0.0504)	0.8250 (± 0.0394)	0.8296 (± 0.0461)	0.8324 (± 0.0591)	0.8426 (± 0.0541)	0.8375 (± 0.0564)	0.8355 (± 0.0482)	**0.8511 (± 0.0432)**	0.8456 (± 0.0429)
sick	3021 (38)	0.9985 (± 0.0009)	0.9985 (± 0.0009)	0.9400 (± 0.0288)	0.9981 (± 0.0010)	0.9967 (± 0.0019)	0.9520 (± 0.0224)	0.9473 (± 0.0315)	0.9512 (± 0.0204)	0.9983 (± 0.0009)	**0.9990 (± 0.0008)**	0.9987 (± 0.0011)
krvskp	3 (3)	0.9998 (± 0.0003)	0.9998 (± 0.0004)	0.9900 (± 0.0040)	0.9995 (± 0.0008)	0.9990 (± 0.0011)	0.9955 (± 0.0028)	0.9998 (± 0.0003)	**0.9999 (± 0.0002)**	0.9994 (± 0.0010)	0.9995 (± 0.0007)	0.9997 (± 0.0005)
LEDdisplaydomain7digit	125921 (40496)	0.9526 (± 0.0133)	0.9531 (± 0.0117)	0.9335 (± 0.0218)	0.9569 (± 0.0132)	0.9539 (± 0.0136)	**0.9584 (± 0.0113)**	0.9538 (± 0.0122)	0.9537 (± 0.0141)	0.9570 (± 0.0122)	0.9530 (± 0.0127)	0.9520 (± 0.0156)
SpeedDating	146607 (40536)	**0.8821 (± 0.0116)**	0.8762 (± 0.0125)	0.7087 (± 0.0187)	0.8794 (± 0.0108)	0.8670 (± 0.0128)	0.8561 (± 0.0138)	0.8355 (± 0.0149)	0.8610 (± 0.0124)	0.8818 (± 0.0129)	0.8734 (± 0.0119)	0.8720 (± 0.0129)
carevaluation	146192 (40664)	**1.0000 (± 0.0000)**	0.9998 (± 0.0007)	0.9927 (± 0.0032)	0.9999 (± 0.0002)	0.9973 (± 0.0016)	0.9922 (± 0.0026)	0.9998 (± 0.0004)	**1.0000 (± 0.0000)**	0.9999 (± 0.0001)	0.9999 (± 0.0002)	0.9998 (± 0.0003)
dna	167140 (40670)	**0.9956 (± 0.0024)**	0.9951 (± 0.0027)	0.9642 (± 0.0067)	0.9949 (± 0.0031)	0.9937 (± 0.0032)	0.9945 (± 0.0033)	0.9919 (± 0.0030)	0.9942 (± 0.0035)	0.9955 (± 0.0025)	0.9954 (± 0.0025)	0.9953 (± 0.0025)
postoperativepatientdata	146210 (40683)	0.4821 (± 0.2380)	0.3948 (± 0.2865)	0.3768 (± 0.1552)	0.3925 (± 0.1394)	0.4631 (± 0.2763)	0.4143 (± 0.1817)	**0.5258 (± 0.1795)**	0.4738 (± 0.1472)	0.4274 (± 0.1235)	0.3500 (± 0.1953)	0.3329 (± 0.1902)
churn	167141 (40701)	**0.9321 (± 0.0230)**	0.9233 (± 0.0188)	0.8479 (± 0.0335)	0.9227 (± 0.0213)	0.9171 (± 0.0216)	0.8624 (± 0.0201)	0.9155 (± 0.0198)	0.9013 (± 0.0225)	0.9205 (± 0.0245)	0.9238 (± 0.0202)	0.9313 (± 0.0139)
yeast	145793 (40733)	0.8586 (± 0.0216)	0.8590 (± 0.0217)	0.8346 (± 0.0300)	0.8550 (± 0.0207)	0.8519 (± 0.0228)	0.8418 (± 0.0227)	0.8483 (± 0.0248)	0.8555 (± 0.0195)	0.8564 (± 0.0221)	**0.8591 (± 0.0225)**	0.8565 (± 0.0242)
Satellite	167211 (40900)	0.9965 (± 0.0033)	0.9909 (± 0.0167)	0.9275 (± 0.0581)	0.9840 (± 0.0260)	0.9832 (± 0.0265)	0.9923 (± 0.0115)	0.9956 (± 0.0037)	0.9917 (± 0.0117)	0.9761 (± 0.0423)	**0.9975 (± 0.0018)**	0.9964 (± 0.0048)
MiceProtein	146800 (40966)	1.0000 (± 0.0000)	1.0000 (± 0.0001)	0.9991 (± 0.0019)	0.9999 (± 0.0001)	0.9999 (± 0.0002)	0.9997 (± 0.0008)	0.9996 (± 0.0008)	**1.0000 (± 0.0000)**	1.0000 (± 0.0001)	**1.0000 (± 0.0000)**	**1.0000 (± 0.0000)**
car	146821 (40975)	**1.0000 (± 0.0000)**	1.0000 (± 0.0000)	0.9927 (± 0.0032)	1.0000 (± 0.0001)	0.9995 (± 0.0005)	0.9922 (± 0.0027)	0.9998 (± 0.0004)	**1.0000 (± 0.0000)**	1.0000 (± 0.0001)	**1.0000 (± 0.0000)**	**1.0000 (± 0.0000)**
mfeatpixel	146824 (40979)	0.9996 (± 0.0004)	0.9996 (± 0.0004)	0.9976 (± 0.0024)	0.9991 (± 0.0007)	0.9989 (± 0.0011)	0.9975 (± 0.0015)	0.9981 (± 0.0013)	0.9996 (± 0.0003)	0.9995 (± 0.0004)	0.9996 (± 0.0005)	**0.9997 (± 0.0003)**
Australian	146818 (40981)	0.9429 (± 0.0199)	0.9423 (± 0.0214)	0.9216 (± 0.0278)	0.9399 (± 0.0178)	0.9385 (± 0.0199)	0.9311 (± 0.0244)	0.8269 (± 0.1744)	0.9289 (± 0.0187)	0.9403 (± 0.0201)	**0.9468 (± 0.0197)**	0.9433 (± 0.0206)
steelplatesfault	146817 (40982)	0.9712 (± 0.0047)	0.9687 (± 0.0061)	0.9245 (± 0.0126)	0.9660 (± 0.0060)	0.9637 (± 0.0076)	0.9325 (± 0.0088)	0.9442 (± 0.0081)	0.9499 (± 0.0089)	0.9687 (± 0.0038)	**0.9788 (± 0.0032)**	0.9783 (± 0.0033)
wilt	146820 (40983)	0.9942 (± 0.0110)	0.9931 (± 0.0078)	0.8539 (± 0.0447)	0.9929 (± 0.0066)	0.9856 (± 0.0168)	0.9751 (± 0.0111)	0.9343 (± 0.0373)	0.9937 (± 0.0050)	0.9909 (± 0.0103)	**0.9950 (± 0.0077)**	0.9946 (± 0.0087)
segment	146822 (40984)	0.9967 (± 0.0012)	0.9962 (± 0.0016)	0.9867 (± 0.0037)	0.9958 (± 0.0018)	0.9957 (± 0.0014)	0.9853 (± 0.0047)	0.9860 (± 0.0051)	0.9915 (± 0.0035)	0.9961 (± 0.0016)	**0.9974 (± 0.0012)**	0.9972 (± 0.0013)
climatemodelsimulationcrashes	146819 (40994)	**0.9596 (± 0.0548)**	0.9538 (± 0.0584)	0.8629 (± 0.1091)	0.9371 (± 0.0602)	0.9155 (± 0.0693)	0.9509 (± 0.0655)	0.9024 (± 0.1109)	0.9474 (± 0.0696)	0.9506 (± 0.0569)	0.9561 (± 0.0631)	0.9576 (± 0.0622)
sonar	39 (40)	0.9536 (± 0.0197)	0.9637 (± 0.0194)	0.9151 (± 0.0602)	0.9453 (± 0.0345)	0.9187 (± 0.0580)	0.8667 (± 0.0554)	0.8881 (± 0.0461)	**0.9685 (± 0.0224)**	0.9506 (± 0.0330)	0.9580 (± 0.0222)	0.9663 (± 0.0195)
jasmine	168911 (41143)	0.8879 (± 0.0185)	0.8825 (± 0.0201)	0.8519 (± 0.0257)	0.8726 (± 0.0171)	0.8849 (± 0.0169)	0.8496 (± 0.0159)	0.8470 (± 0.0215)	0.8584 (± 0.0197)	0.8794 (± 0.0176)	**0.8904 (± 0.0138)**	0.8861 (± 0.0166)
philippine	190410 (41145)	0.8772 (± 0.0143)	0.8632 (± 0.0154)	0.7735 (± 0.0120)	0.8660 (± 0.0148)	0.8437 (± 0.0162)	0.8019 (± 0.0218)	0.7991 (± 0.0226)	0.7974 (± 0.0205)	0.8643 (± 0.0153)	**0.9751 (± 0.0043)**	0.9685 (± 0.0055)
sylvine	168912 (41146)	0.9926 (± 0.0034)	0.9882 (± 0.0043)	0.8931 (± 0.0096)	0.9883 (± 0.0045)	0.9817 (± 0.0044)	0.9660 (± 0.0062)	0.9746 (± 0.0056)	0.9731 (± 0.0065)	0.9879 (± 0.0034)	**0.9972 (± 0.0017)**	0.9967 (± 0.0018)
glass	40 (41)	0.9627 (± 0.0197)	0.9674 (± 0.0201)	0.8860 (± 0.0582)	0.9435 (± 0.0174)	0.9586 (± 0.0203)	0.8870 (± 0.0422)	0.8656 (± 0.0389)	0.9109 (± 0.0482)	0.9366 (± 0.0365)	**0.9749 (± 0.0200)**	0.9709 (± 0.0281)
haberman	42 (43)	**0.7467 (± 0.0740)**	0.7007 (± 0.0832)	0.6117 (± 0.1130)	0.6804 (± 0.0918)	0.6779 (± 0.0870)	0.6687 (± 0.0933)	0.6321 (± 0.1074)	0.6576 (± 0.0553)	0.6829 (± 0.1120)	0.7021 (± 0.0857)	0.6893 (± 0.0864)
analcatdata_boxing1	3540 (448)	0.9076 (± 0.0796)	0.8753 (± 0.1232)	0.7841 (± 0.1073)	0.6179 (± 0.0885)	0.8646 (± 0.1051)	**0.9378 (± 0.0627)**	0.8221 (± 0.2038)	0.8821 (± 0.1180)	0.6241 (± 0.2107)	0.8990 (± 0.1038)	0.8712 (± 0.0928)
spambase	43 (44)	**0.9913 (± 0.0064)**	0.9893 (± 0.0064)	0.9550 (± 0.0098)	0.9904 (± 0.0065)	0.9876 (± 0.0058)	0.9718 (± 0.0092)	0.9819 (± 0.0076)	0.9786 (± 0.0078)	0.9901 (± 0.0061)	0.9909 (± 0.0065)	0.9904 (± 0.0062)
irish	3543 (451)	**1.0000 (± 0.0000)**	0.9989 (± 0.0025)	0.9999 (± 0.0003)	**1.0000 (± 0.0000)**	**1.0000 (± 0.0000)**	**1.0000 (± 0.0000)**	**1.0000 (± 0.0000)**	**1.0000 (± 0.0000)**	0.9358 (± 0.0641)	**1.0000 (± 0.0000)**	**1.0000 (± 0.0000)**
GesturePhaseSegmentationProcessed	14969 (4538)	0.9425 (± 0.0065)	0.9182 (± 0.0089)	0.8380 (± 0.0108)	0.9164 (± 0.0070)	0.8968 (± 0.0074)	0.7616 (± 0.0091)	0.7836 (± 0.0093)	0.7943 (± 0.0037)	0.9193 (± 0.0068)	**0.9495 (± 0.0054)**	0.9407 (± 0.0082)
analcatdata_authorship	3549 (458)	1.0000 (± 0.0001)	1.0000 (± 0.0001)	1.0000 (± 0.0001)	1.0000 (± 0.0000)	1.0000 (± 0.0001)	1.0000 (± 0.0000)	1.0000 (± 0.0000)	**1.0000 (± 0.0000)**	**1.0000 (± 0.0000)**	**1.0000 (± 0.0000)**	**1.0000 (± 0.0000)**
analcatdata_dmft	3560 (469)	0.5889 (± 0.0404)	0.5835 (± 0.0383)	0.5464 (± 0.0286)	0.5834 (± 0.0373)	**0.6078 (± 0.0419)**	0.5792 (± 0.0294)	0.5710 (± 0.0393)	0.5907 (± 0.0343)	0.5881 (± 0.0325)	0.5951 (± 0.0294)	0.5943 (± 0.0260)
splice	45 (46)	**0.9957 (± 0.0032)**	0.9952 (± 0.0030)	0.9764 (± 0.0049)	0.9944 (± 0.0036)	0.9943 (± 0.0027)	0.9933 (± 0.0033)	0.9918 (± 0.0032)	0.9948 (± 0.0038)	0.9949 (± 0.0021)	0.9955 (± 0.0030)	0.9947 (± 0.0033)
profb	3561 (470)	**0.8062 (± 0.0620)**	0.7978 (± 0.0483)	0.5649 (± 0.0583)	0.7255 (± 0.0740)	0.6460 (± 0.0664)	0.6538 (± 0.0455)	0.6604 (± 0.0508)	0.6347 (± 0.0577)	0.5674 (± 0.0370)	0.6550 (± 0.0560)	0.6850 (± 0.0533)
tae	47 (48)	0.8113 (± 0.0769)	**0.8436 (± 0.0741)**	0.7170 (± 0.0714)	0.8005 (± 0.0689)	0.8131 (± 0.0725)	0.6678 (± 0.0982)	0.6421 (± 0.1195)	0.7020 (± 0.0664)	0.8313 (± 0.0742)	0.8254 (± 0.0639)	0.8300 (± 0.0783)
heartc	48 (49)	0.9194 (± 0.0526)	0.9153 (± 0.0253)	0.9020 (± 0.0391)	0.9178 (± 0.0316)	0.9171 (± 0.0234)	0.9138 (± 0.0263)	0.9112 (± 0.0422)	0.9128 (± 0.0368)	0.9036 (± 0.0536)	0.9212 (± 0.0306)	**0.9230 (± 0.0295)**
labor	4 (4)	0.9750 (± 0.0527)	0.9708 (± 0.0623)	0.9542 (± 0.1084)	0.9375 (± 0.1062)	0.9833 (± 0.0527)	0.9875 (± 0.0395)	0.9750 (± 0.0791)	0.9833 (± 0.0527)	0.9875 (± 0.0395)	**1.0000 (± 0.0000)**	0.9875 (± 0.0395)
tictactoe	49 (50)	**1.0000 (± 0.0000)**	**1.0000 (± 0.0000)**	0.9993 (± 0.0008)	**1.0000 (± 0.0000)**	0.9983 (± 0.0012)	0.9973 (± 0.0019)	0.9987 (± 0.0014)	**1.0000 (± 0.0000)**	**1.0000 (± 0.0000)**	**1.0000 (± 0.0000)**	**1.0000 (± 0.0000)**
hearth	50 (51)	0.9043 (± 0.0517)	0.9078 (± 0.0357)	0.8973 (± 0.0430)	0.8901 (± 0.0373)	0.9108 (± 0.0309)	0.9115 (± 0.0322)	0.8800 (± 0.0732)	0.9028 (± 0.0327)	0.8698 (± 0.0493)	**0.9126 (± 0.0415)**	0.9112 (± 0.0350)
vehicle	53 (54)	0.9737 (± 0.0062)	0.9398 (± 0.0132)	0.9031 (± 0.0122)	0.9323 (± 0.0110)	0.9320 (± 0.0114)	0.9484 (± 0.0118)	0.9342 (± 0.0197)	0.9663 (± 0.0100)	0.9347 (± 0.0154)	**0.9750 (± 0.0054)**	0.9735 (± 0.0059)
hepatitis	54 (55)	0.8399 (± 0.1399)	0.8619 (± 0.1135)	0.8206 (± 0.1377)	0.7939 (± 0.1213)	0.8704 (± 0.1159)	0.8516 (± 0.1167)	0.7974 (± 0.1451)	0.7975 (± 0.1248)	0.8636 (± 0.1093)	**0.8781 (± 0.1160)**	0.8718 (± 0.1112)
ionosphere	145984 (59)	0.9880 (± 0.0125)	0.9762 (± 0.0210)	0.9278 (± 0.0546)	0.9766 (± 0.0171)	0.9785 (± 0.0203)	0.9181 (± 0.0565)	0.9714 (± 0.0232)	0.9803 (± 0.0203)	0.9811 (± 0.0199)	**0.9894 (± 0.0136)**	0.9880 (± 0.0149)
iris	59 (61)	**0.9987 (± 0.0042)**	0.9947 (± 0.0129)	**0.9987 (± 0.0042)**	0.9827 (± 0.0310)	0.9967 (± 0.0072)	**0.9987 (± 0.0042)**	0.9493 (± 0.0687)	**0.9987 (± 0.0042)**	0.9927 (± 0.0152)	**0.9987 (± 0.0042)**	**0.9987 (± 0.0042)**
cylinderbands	14954 (6332)	**0.9238 (± 0.0477)**	0.9067 (± 0.0563)	0.8221 (± 0.0691)	0.9141 (± 0.0549)	0.9086 (± 0.0493)	0.8229 (± 0.0717)	0.8659 (± 0.0752)	0.8793 (± 0.0672)	0.8839 (± 0.0720)	0.8983 (± 0.0577)	0.8936 (± 0.0656)
visualizing_environmental	3602 (736)	0.7311 (± 0.1269)	0.6978 (± 0.1428)	0.6778 (± 0.1487)	0.6844 (± 0.1652)	0.7256 (± 0.1486)	0.7231 (± 0.1844)	0.6856 (± 0.1932)	0.7461 (± 0.1628)	0.6969 (± 0.1737)	0.7583 (± 0.1434)	**0.7611 (± 0.1363)**
fri_c0_100_5	3620 (754)	0.9433 (± 0.0715)	0.9548 (± 0.0597)	0.9472 (± 0.0413)	0.9343 (± 0.0625)	0.9180 (± 0.1034)	0.9060 (± 0.0806)	0.7488 (± 0.1836)	0.9432 (± 0.0580)	0.9428 (± 0.0731)	0.9427 (± 0.0676)	**0.9592 (± 0.0508)**
rabe_266	3647 (782)	0.9944 (± 0.0119)	0.9972 (± 0.0088)	0.9901 (± 0.0190)	0.9831 (± 0.0228)	0.9944 (± 0.0117)	0.9887 (± 0.0196)	0.9633 (± 0.0598)	**1.0000 (± 0.0000)**	0.9931 (± 0.0176)	0.9972 (± 0.0088)	0.9972 (± 0.0088)
visualizing_livestock	3731 (867)	0.8391 (± 0.1330)	0.8417 (± 0.1389)	0.8191 (± 0.1127)	0.5000 (± 0.0000)	0.8139 (± 0.1533)	**0.8900 (± 0.0837)**	0.8685 (± 0.1182)	0.8085 (± 0.1472)	0.7658 (± 0.1936)	0.8512 (± 0.1329)	0.8530 (± 0.1929)
pollen	3735 (871)	0.4921 (± 0.0215)	0.4782 (± 0.0234)	0.4927 (± 0.0196)	0.4906 (± 0.0359)	0.4824 (± 0.0157)	0.4871 (± 0.0119)	**0.5024 (± 0.0166)**	0.4980 (± 0.0154)	0.4856 (± 0.0252)	0.4845 (± 0.0197)	0.4937 (± 0.0213)
analcatdata_chlamydia	3739 (875)	0.9701 (± 0.0505)	0.9764 (± 0.0403)	0.9090 (± 0.0778)	0.5965 (± 0.2410)	0.9764 (± 0.0499)	0.9639 (± 0.0503)	0.9389 (± 0.1012)	0.9451 (± 0.0845)	0.8479 (± 0.2554)	**0.9812 (± 0.0422)**	0.9701 (± 0.0505)
transplant	3748 (885)	**1.0000 (± 0.0000)**	**1.0000 (± 0.0000)**	**1.0000 (± 0.0000)**	0.9963 (± 0.0119)	**1.0000 (± 0.0000)**	0.9975 (± 0.0079)	**1.0000 (± 0.0000)**	**1.0000 (± 0.0000)**	**1.0000 (± 0.0000)**	**1.0000 (± 0.0000)**	**1.0000 (± 0.0000)**
fri_c3_100_5	3779 (916)	0.9468 (± 0.0717)	0.9223 (± 0.0733)	0.8871 (± 0.0760)	0.8932 (± 0.1120)	0.9345 (± 0.0724)	0.7375 (± 0.1229)	0.6325 (± 0.2052)	0.9465 (± 0.0751)	0.9507 (± 0.0541)	**0.9592 (± 0.0606)**	0.9548 (± 0.0735)
socmob	3797 (934)	**0.9917 (± 0.0051)**	0.9861 (± 0.0101)	0.9818 (± 0.0150)	0.9877 (± 0.0071)	0.9823 (± 0.0097)	0.9839 (± 0.0068)	0.9833 (± 0.0092)	0.9757 (± 0.0116)	0.9906 (± 0.0039)	0.9905 (± 0.0072)	0.9916 (± 0.0062)
autos	9 (9)	0.9466 (± 0.0468)	0.9619 (± 0.0191)	0.8634 (± 0.0420)	0.9593 (± 0.0446)	0.9608 (± 0.0191)	0.8833 (± 0.0504)	0.8889 (± 0.0454)	0.9149 (± 0.0346)	0.9408 (± 0.0383)	**0.9779 (± 0.0193)**	0.9750 (± 0.0174)
