# Supplementary material for: Accurate predictions on small data with a tabular foundation model
Source: Nature. 2025 Jan 8;637(8045):319–26. doi: 10.1038/s41586-024-08328-6 (PMC11711098; doi:10.1038/s41586-024-08328-6)
Supplement: Supplementary file 2 — Meta-information on development datasets: meta-information of the development dataset is used to validate the performance of our models for regression and classification. [file 41586_2024_8328_MOESM2_ESM.pdf]

Supplementary Table 5: **List of development datasets used to validate classification tasks.** We only take the first 10000 rows of all datasets and drop datasets with more than 500 features or 10 classes to keep the datasets similar to our test dataset distribution. We detected overlap with the test set by checking for duplicate names and meta-data characteristics (rows, features, missing values).

Dataset Name	OpenML ID	Features	Samples	Targets	Categorical Feats.	Numerical Feats.
adaprior	1037	14	4562	2	8	6
allbp	40707	29	3772	3	23	6
analcatdata_asbestos	459	3	83	2	2	1
analcatdata_cyyoung8092	465	10	97	2	3	7
ar3	1060	29	63	2	0	29
ar6	1064	29	101	2	0	29
baseball	185	16	1340	3	1	15
birds	40588	278	645	2	2	276
blogger	1463	5	100	2	5	0
breast-tissue	1465	9	106	6	0	9
cjs	473	34	2796	6	2	32
clean1	40665	168	476	2	0	168
cleveland-nominal	40711	7	303	5	7	0
compas-two-years	42192	13	5278	2	6	7
confidence	468	3	72	6	0	3
corral	40669	6	160	2	6	0
delta_ailerons	803	5	7129	2	0	5
dermatology	35	34	366	6	33	1
eye_movements	44130	20	7608	2	0	20
GAMETES_Epistasis	40646	20	1600	2	20	0
grub-damage	338	8	155	4	6	2
heart-c	49	13	303	2	7	6
heart-h	51	13	294	2	7	6
heart-statlog	53	13	270	2	0	13
heloc	45026	22	10000	2	0	22
hill-valley	1479	100	1212	2	0	100
ionosphere	59	34	351	2	0	34
JapaneseVowels	375	14	9961	9	0	14
jungle_chess_2pcs_endgame_panther_lion	40997	46	4704	3	26	20
led24	40677	24	3200	10	24	0
mc1	1056	38	9466	2	0	38
Midwest_Survey_nominal	42532	27	2778	10	27	0
mw1	1071	37	403	2	0	37
national-longitudinal-survey-binary	43892	16	4908	2	7	9
olindda_outliers	42793	2	75	4	0	2
page-blocks	30	10	5473	5	0	10
penguins	42585	6	344	3	2	4
regime_alimentaire	42172	19	202	2	16	3
ringnorm	1496	20	7400	2	0	20
rl	44160	12	4970	2	7	5
rmftsa_sleepdata	679	2	1024	4	0	2
scene	312	299	2407	2	5	294
Smartphone-Based_Recognition	4153	66	180	6	0	66
squash-stored	340	24	52	3	3	21
synthetic_control	377	60	600	6	0	60
teachingAssistant	1115	6	151	3	4	2
thyroid-allbp	40474	26	2800	5	20	6
thyroid-ann	40497	21	3772	3	0	21
Titanic	40704	3	2201	2	0	3
tokyo1	40705	44	959	2	2	42
Tour-and-Travels-Churn	45545	6	954	2	4	2
user-knowledge	1508	5	403	5	0	5
volcanoes-a1	1527	3	3252	5	0	3
vote	56	16	435	2	16	0
waveform-5000	60	40	5000	3	0	40
wholesale-customers	1511	8	440	2	1	7
xd6	40693	9	973	2	9	0

Supplementary Table 6: **List of development datasets used to validate regression tasks.** We only take the first 10000 rows of all datasets and drop datasets with more than 500 features or 10 classes to keep the datasets similar to our test dataset distribution. We detected overlap with the test set by checking for duplicate names and meta-data characteristics (rows, features, missing values).

Dataset Name	OpenML ID	Features	Samples	Targets	Categorical Feats.	Numerical Feats.
AAPL_stock_price_2021_2022	43878	5	346	334	0	5
ames_housing	43926	80	2930	1032	46	34
analcatdata_apnea3	555	3	450	159	2	1
analcatdata_birthday	456	3	365	336	3	0
analcatdata_gviolence	518	8	74	73	0	8
analcatdata_michiganacc	551	3	108	26	2	1
analcatdata_supreme	44055	7	4052	10	5	2
arsenic-male-bladder	482	4	559	14	1	3
autoMpg	196	7	398	129	3	4
autoPrice	207	15	159	145	0	15
balloon	512	1	2001	365	0	1
bodyfat	560	14	252	176	0	14
boston_corrected	543	19	506	455	3	16
chatfield_4	695	12	235	211	0	12
chscase_vine2	689	2	468	160	0	2
cloud	210	5	108	94	2	3
cocomo_numeric	1051	16	60	49	15	1
CPMP-2015-runtime-regression	41928	23	2108	1161	1	22
cps_85_wages	534	10	534	238	7	3
cpu_act	44132	21	8192	56	0	21
cpu_small	227	12	8192	56	0	12
crimecommunitynums	41968	126	1994	46	0	126
crimecommunitynums2	41969	126	1994	46	0	126
debutanizer	23516	7	2394	2337	0	7
DEE	42360	6	365	365	0	6
delta_elevators	198	6	9517	26	0	6
ELE-2	42362	4	1056	1011	0	4
fishcatch	232	7	158	101	2	5
GeographicalOriginalofMusic	4544	117	1059	1057	0	117
kc1-numeric	1070	94	145	24	0	94
kdd_coil_1	567	11	316	166	3	8
kidney	553	6	76	20	3	3
laser	42364	4	993	191	0	4
mauna-loa-atmospheric-co2	41187	6	2225	581	1	5
meta	566	21	528	436	2	19
MIP-2016-PAR10-regression	41938	144	1090	924	1	143
mtp	405	202	4450	800	0	202
plasma_retinol	511	13	315	257	3	10
pm10	522	7	500	117	0	7
pollen	529	4	3848	3784	0	4
puma32H	308	32	8192	7861	0	32
QSAR_fish_toxycity	44028	6	908	827	0	6
rabe_265	660	6	51	47	0	6
rabe_266	663	2	120	96	0	2
RAM_price	40601	1	333	219	0	1
residential_building	42366	108	372	117	0	108
S1	42110	2	5000	15	0	2
stock_fardamento02	42545	6	6277	113	1	5
treasury	42367	15	1049	570	0	15
triazines	206	60	186	102	0	60
visualizing_soil	44056	4	8641	40	1	3
weather_ankara	42368	9	321	251	0	9
weather_izmir	42369	9	1461	489	0	9
wind	503	14	6574	779	0	14
